# A UK population-based case-control study of blood tests before cancer diagnosis in patients with non-specific abdominal symptoms

**DOI:** 10.1038/s41416-024-02936-9

**Published:** 2025-01-11

**Authors:** Meena Rafiq, Becky White, Matthew Barclay, Gary Abel, Cristina Renzi, Georgios Lyratzopoulos

**Affiliations:** 1 Epidemiology of Cancer Healthcare & Outcomes (ECHO) Group, Department of Behavioural Science, Institute of Epidemiology and Health Care (IEHC), UCL, London, UK; 2 Department of General Practice and Primary Care, University of Melbourne, Melbourne, Australia; 3 University of Exeter Medical School, Exeter, UK; 4 Faculty of Medicine, University Vita-Salute San Raffaele, Milan, Italy

**Keywords:** Cancer epidemiology, Pathology, Digestive signs and symptoms, Cancer epidemiology

## Abstract

**Background:**

Abnormal results in commonly used primary care blood tests could be early markers of cancer in patients presenting with non-specific abdominal symptoms.

**Methods:**

Using linked data from the UK Clinical Practice Research Datalink (CPRD) and national cancer registry we compared blood test use and abnormal results from the 24-months pre-diagnosis in 10,575 cancer patients (any site), and 52,875 matched-controls aged ≥30 presenting, with abdominal pain or bloating to primary care.

**Results:**

Cancer patients had two-fold increased odds of having a blood test (odds ratio(OR):1.51–2.29) and 2-3-fold increased odds of having an abnormal blood test result (OR:2.42–3.30) in the year pre-diagnosis compared to controls. Raised inflammatory markers were the most common abnormality (74–79% of tested cases). Rates of blood test use and abnormal results progressively increased from 7 months pre-diagnosis in cancer patients, with relatively small corresponding increases in symptomatic controls. In cancer patients, the largest increases from baseline were raised platelets in males with abdominal pain (increased 33-fold), raised white blood cell count in males with abdominal bloating (increased 37-fold) and low albumin in females with either symptom (increased 22–41 fold).

**Conclusions:**

Common blood test abnormalities are early signals of cancer in some individuals with non-specific abdominal symptoms and could support expedited cancer diagnosis.

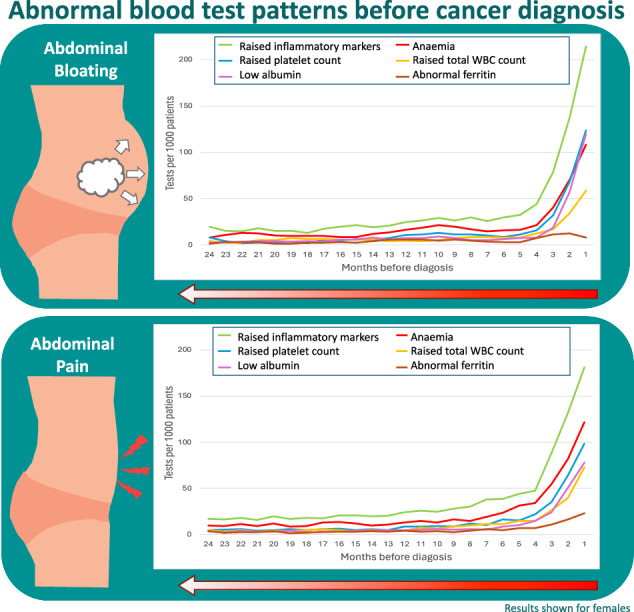

## Introduction

Most patients with cancer are diagnosed after presenting with symptoms to primary care[1, 2]. Approximately half of these patients have well recognised ‘red flag’ symptoms, which align with existing international urgent cancer referral pathways to support faster diagnosis and treatment [3–6]. The other half of patients pose a diagnostic challenge. They present with non-specific symptoms that overlap with many commonly encountered, often benign, conditions, making their cancer hard to identify early [7, 8]. These patients frequently experience complex care pathways, with multiple consultations and investigations before their cancer is eventually detected [9–11]. They are therefore more likely to be diagnosed with advanced stage cancer [9] and via emergency presentations [9, 12], which are associated with poorer outcomes [13–15] and worse patient experience [16].

Two non-specific symptoms that present a particular dilemma for general practitioners (GPs) are abdominal pain and bloating. These symptoms are commonly encountered in the population, with one in three people experiencing bloating in the last 30 days and one in five having abdominal pain [17], and many of these patients will present to primary care [18] (abdominal symptoms have been reported to feature in 10% of adult primary care consultations [19]). For the most part, these symptoms are attributable to a wide range of largely benign causes, but in a small proportion of patients they represent presenting features of cancer of different sites [20–22]. Research is needed to understand if opportunities for earlier diagnosis exist in cancer patients who present with these symptoms and how these patients can be better identified in primary care. Such information could improve earlier-stage cancer diagnosis, survival and patient outcomes [23].

Examining commonly used blood tests could reveal potential opportunities for earlier cancer diagnosis. Primary care requests for several blood tests, particularly acute phase reactants (APR), increase in the year before cancer diagnosis [24–27], with abnormal results (values occurring outside standard laboratory reference ranges for the population) occurring several months before cancer diagnosis for a number of blood tests and cancer sites [24, 25, 28]. Abnormalities in these blood tests may be early signals or predictors of some cancers [24, 29, 30]. In patients presenting to primary care with weight loss, another non-specific symptom, blood test abnormalities were predictive of as-yet-undetected cancer and could potentially help GPs to identify the subgroup of patients who should be prioritised for further cancer investigation [31]. The discriminative value of abnormal blood tests in identifying patients with abdominal pain or bloating who warrant further investigation for possible cancer remains unclear.

This study aimed to determine when blood test use first starts to increase prior to cancer diagnosis (of any cancer site) among patients who presented to primary care with abdominal pain or bloating, comparing patients with and without as-yet-undetected cancer, and to identify if abnormalities in commonly used blood tests could be early signals of cancer (focusing on anaemia and APR tests).

## Methods

### Design and setting

We conducted a nested, matched case-control study, including patients identified from two symptomatic cohorts of patients presenting to primary care between 1st January 2007 and 31st October 2016 with either new onset abdominal pain (cohort 1) or new onset abdominal bloating (cohort 2). Primary care data from the UK Clinical Practice Research Datalink (CPRD) [32] was used to collect coded patient information on presenting symptoms, blood tests and demographics. This was linked to the National Cancer Registration and Analysis Service dataset (NCRAS) (with follow up data available to 31st October 2017) and Hospital Episode Statistic (HES) Admitted Patient Care (APC) data for cancer diagnoses. Linked Index of Multiple Deprivation (IMD) quintile data were used to provide an indication of patient socioeconomic status.

### Defining the symptomatic cohorts

The two symptomatic cohorts comprised all patients actively registered at a CPRD practice, aged ≥30 years, with at least one valid episode of either abdominal pain or bloating coded in CPRD during the study period (see Supplementary Table S1 for code lists). Comprehensive symptom code lists, cross checked by three clinicians, were used to identify recorded events of abdominal pain or bloating to ensure accurate identification. We defined active CPRD follow up as starting from when a patient is registered for at least a year at a practice considered up to standard regarding data quality and ending when a patient dies, leaves the practice, or practice data stops being collected. To ensure only new symptomatic presentations were included, a valid symptom episode was defined as one recorded while the patient had been registered at the practice for at least 365 days before the episode, with no other episode of the same symptom recorded during this period. For each patient, the date of the first valid episode of abdominal pain or bloating was identified and set as the ‘first symptom date’ and the date of any further presentations with abdominal pain or bloating in the 12 months after this date were extracted as repeat presentations. Patients with a valid episode of both abdominal pain and bloating could appear in both cohorts (with different first symptom dates). Patients were excluded if they were not eligible for linkage to the cancer registry or if they had a previous record of any cancer diagnosis (recorded in NCRAS / CPRD / HES) prior to the first symptom date.

### Identification of cancer cases and controls

Each patient in the symptomatic cohorts was followed up for 12 months after their first symptom date to identify any new cancer diagnoses recorded in the national cancer registry (excluding non-melanoma skin cancer and benign brain tumours as is standard practice) [33]. Therefore, all cancer cases in the study arise from these symptomatic cohorts, followed up over a 12-month period, as is customary for identifying cases related to presenting symptoms. All patients with a cancer diagnosis in this period were selected as cases. The earliest date of cancer diagnosis was set as the ‘index date’ and the cancer site was recorded. As primary care activity (in the form of consultations and blood test requests) is expected to increase following a symptomatic presentation, a comparison group of symptomatic controls, who presented with the same symptom but did not develop cancer, was used. This comparison enabled identification of any differentiating trends between patients with and without as-yet-undetected cancer who present with the same symptom in primary care. To do this, from the remaining pool of patients in each symptomatic cohort who were not diagnosed with cancer, five controls per case were selected by individual matching based on age ( ± 1 year), sex and calendar time of the symptom date ( ± 30 days). Controls were selected at random using a random number generator from the pool of eligible matches for each case. An index date was assigned to each control corresponding to the date of cancer diagnosis in their matched case.

### Defining the explanatory variables

Alongside haemoglobin, a well-recognised predictor of possible cancer, five acute phase reactant (APR) blood tests were selected a priori for inclusion: inflammatory markers (comprising erythrocyte sedimentation rate (ESR) and C reactive protein (CRP)), platelet count and white blood cell count (WBC) (whose values all increase as part of the acute phase response), albumin (whose values decrease during the acute phase response), and ferritin concentration (which can both increase in the acute phase response or decrease as a result of anaemia). Haemoglobin concentration, platelet count and white blood cell count form part of a full blood count (FBC) panel and albumin forms part of a liver function test (LFT) panel. The date and result of all relevant tests in the 24 months before the cancer diagnosis/index date were extracted (as is standard practice for cancer diagnostic window studies [34]) and cleaned using the following steps: 1. select the most frequently used units and reference ranges for each test (and where required converting results reported in other units), 2. exclude results with biologically implausible values, 3. remove duplicate tests on the same day (selecting the mean value of the results if there are duplicates), 4. classify each result as normal or abnormal based on standard laboratory reference ranges.

### Statistical analysis

All analyses were conducted for the two study cohorts separately (patients with abdominal pain or abdominal bloating) and stratifying by sex.

Conditional logistic regression analyses were used to compare characteristics in cases and controls and examine associations between cancer diagnosis and a.) repeat primary care consultations for abdominal pain or bloating b.) blood test requests or c.) related blood test abnormalities at any point in the preceding year. Analyses were conducted for all APR tests combined as a composite variable and for each of the test types individually.

To analyse longitudinal trends in primary care consultations for the abdominal symptoms and blood test use prior to cancer diagnosis, mixed-effects Poisson regression models were used. We estimated monthly consultation rates (for abdominal pain or abdominal bloating) and blood test request rates with 95% confidence intervals for the 24 months preceding cancer diagnosis in cases. As primary healthcare use and diagnostic activity are expected to increase following a consult for a symptom, comparative analyses were conducted in a symptomatic control group who presented to primary care with symptoms at the same time as cases ( ± 30 days) but did not have cancer to differentiate any increases limited to the cancer group. A random intercept for matching set was included in the model to account for the case-control design. Monthly Rate Ratios (RR) were estimated comparing consultation rates and blood test request rates in each of the 24 months to the baseline rate at 2 years before the index date, in cases and controls separately. To identify the timing of the inflection point where diagnostic activity increased prior to cancer diagnosis we used the method previously described by Moullet et al. and Price et al. [34, 35]. Briefly, a case-only analysis was used to run a series of Poisson regression models with sequential monthly inflection points to select the model with the best fit for identifying when consultation rates and blood test request rates first start to deviate from the background trend in cancer patients. Analyses were initially conducted considering all APR blood tests combined and then repeated for each individual test type. Additional analyses examined the monthly proportion of cases and controls who had a blood test request in each of the 24 months before the index date, plotting the monthly incident and cumulative percentage of tested patients over time.

To examine the timing of blood test abnormalities before cancer diagnosis within these symptomatic populations, the above analyses were repeated replacing the binary blood test request variable (yes/no) with a blood test abnormality variable (yes/no) considering APR tests combined followed by each of the six blood tests individually. We estimated when blood test abnormalities first started to increase from the background rate and for which blood tests, comparing whether similar patterns were seen in cases and controls. Following this we calculated the monthly percentage of patients (incident percentage and cumulative percentage over time) who had abnormalities detected before cancer diagnosis/index date, comparing patterns in cases and controls.

## Results

425,549 patients with new onset abdominal pain and 52,321 patients with new abdominal bloating aged ≥30, were identified from the CPRD dataset (6% of patients appeared in both cohorts). Within 12 months of presentation to primary care, 9427 abdominal pain patients (2.21%, 95%CI 2.17–2.26) and 1148 abdominal bloating patients (2.19%, 95%CI 2.07–2.32) were diagnosed with cancer and selected as cases. The distribution of cancer sites diagnosed in these patients has been reported previously [36]. Cases from each symptom cohort were matched to 47,135 and 5740 controls from the larger pool of symptomatic patients, respectively. In both the abdominal pain and bloating cohorts, the mean age at presentation was 67 years (except for abdominal bloating controls where it was 66 years), there was a higher proportion of females compared to males (54% abdominal pain, 68% abdominal bloating), and patients were more likely to be from the least deprived IMD quintile (24–26% in the top IMD quintile vs 14% in the lowest) (Supplementary Table S2).

### Primary care consultations for abdominal pain or bloating

Repeat primary care presentations for the abdominal symptoms were more common in patients subsequently diagnosed with cancer, with 31% of male and 32% of female cases having repeat abdominal pain presentations pre-diagnosis, and 10 and 16% for abdominal bloating (compared to 14 and 15% of controls for abdominal pain and 9% for abdominal bloating) (Supplementary Table S3). Repeat abdominal pain presentations pre-diagnosis were three-fold more likely in cancer patients than controls for both sexes (odds ratio (OR) 2.73, 95%CI 2.54–2.93 males; OR 2.99, 95%CI 2.75–3.25 females). Repeat abdominal bloating presentation were twice as likely in female cancer patients (OR 2.19, 95%CI 1.69–2.86), but no difference was found in males (Supplementary Table S3).

**Fig. 1:**
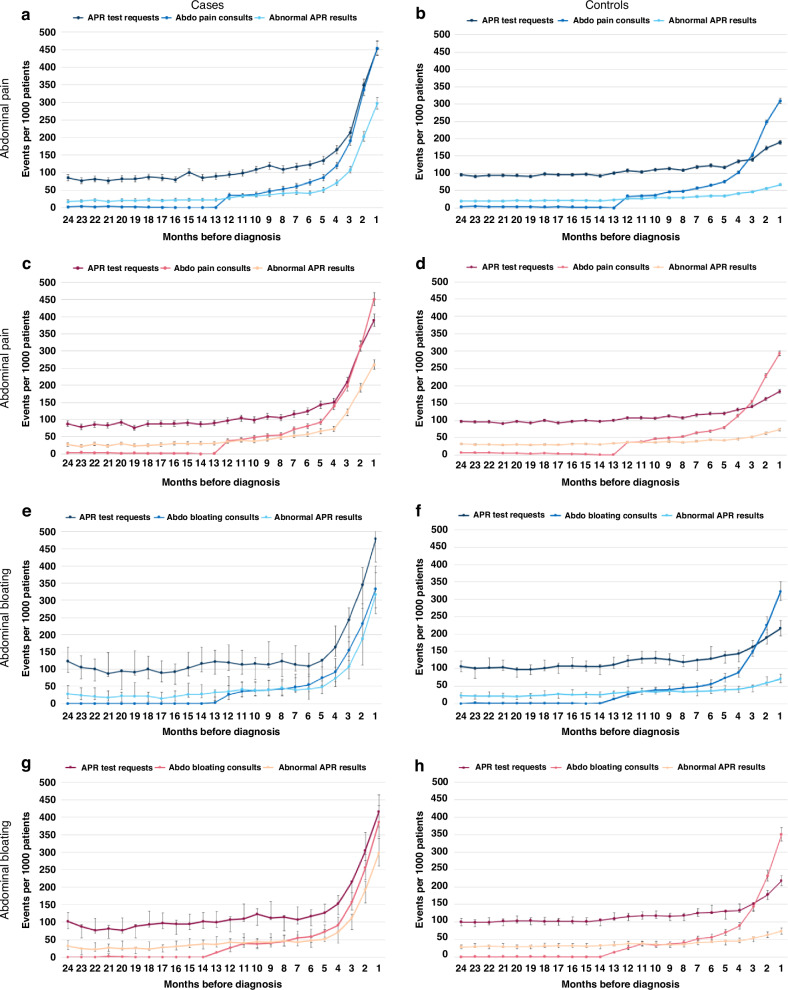
Diagnostic activity in general practice in cases and controls presenting with abdominal pain and bloating in the 24 months before diagnosis/index date. Monthly rates of GP consults for abdominal pain/bloating, APR blood test requests and abnormal APR blood results. Panels **a**, **b**, **e**, **f** show trends in males, panels **c**, **d**, **g**, **h** show trends in females; APR, acute phase response blood tests; abdo, abdominal.

In both symptomatic cancer patients and controls, consultations for abdominal pain or bloating first start to increase from 5 months before diagnosis/index date (Fig. 1). For abdominal bloating, the rate of consultations for this symptom over time was comparable between cancer patients and controls. For abdominal pain however, the monthly rate of consultations was higher in patients with cancer from 5 months before diagnosis, increasing to a peak of 451–455 consultations/1000 patients in the month immediately before diagnosis compared to 293–309 consultations/1000 patients in non-cancer controls (Fig. 1).

### Blood test use

Among cancer patients presenting with either symptom, 73-81% had one or more APR blood tests (platelet, inflammatory marker, white blood cell (WBC), ferritin, albumin) in the year before diagnosis (Table 1). Cancer patients had 1.51–2.29 fold increased odds of having a blood test in the 12 months before diagnosis compared to controls (*p* < 0.001) (Table 1). Similar patterns were seen across all test types, apart from ferritin in females with abdominal bloating where no difference was seen in test use between cases and controls (Table 1). In both cancer patients and symptomatic controls, there was a progressive increase in the monthly proportion of patients with one of these blood tests from 6 months pre-diagnosis, but the magnitude of the increase was much greater in cases, rising from <10% at baseline (-24 months) to 35% in the month preceding diagnosis, while remaining <18% in controls (Supplementary Figure S1).

**Table 1:** Association between cancer diagnosis and blood test requests in the year preceding diagnosis/index date in patients presenting with abdominal pain (*n* = 56562) or bloating (*n* = 6888)

Blood test request	Cancer Patients *n* (% out of all tested patients)	Controls *n* (% out of all tested patients)	Absolute difference	Odds Ratio (95%CI)	*P* value
Males with Abdominal Pain (*n* = 26232)					
	(*n* = 4372)	(*n* = 21860)			
Any APR test	3794 (87%)	16320 (75%)	12%	2.29 (2.09–2.52)	<0.001
Platelet	3564 (82%)	14190 (65%)	17%	2.44 (2.25–2.65)	<0.001
Albumin	3528 (81%)	14903 (68%)	13%	1.99 (1.84–2.16)	<0.001
IM (ESR / CRP)	2390 (55%)	7831 (36%)	19%	2.18 (2.04–2.33)	<0.001
Ferritin	888 (20%)	2555 (12%)	8%	1.95 (1.79–2.12)	<0.001
WBC	3562 (81%)	14213 (65%)	16%	2.42 (2.23–2.63)	<0.001
Haemoglobin	3583 (82%)	14264 (65%)	16%	2.47 (2.28–2.69)	<0.001
Females with Abdominal Pain (*n* = 30330)					
	(*n* = 5055)	(n = 25275)			
Any APR test	4159 (82%)	18438 (73%)	9%	1.75 (1.62–1.90)	<0.001
Platelet	3952 (78%)	16555 (66%)	12%	1.92 (1.78–2.06)	<0.001
Albumin	3739 (74%)	16126 (64%)	10%	1.65 (1.54–1.77)	<0.001
IM (ESR / CRP)	2657 (53%)	9755 (39%)	14%	1.77 (1.67–1.89)	<0.001
Ferritin	1068 (21%)	3611 (14%)	7%	1.63 (1.51–1.76)	<0.001
WBC	3953 (78%)	16597 (66%)	12%	1.90 (1.77–2.05)	<0.001
Haemoglobin	3963 (78%)	16656 (66%)	12%	1.91 (1.77–2.05)	<0.001
Males with Abdominal Bloating (*n* = 2202)					
	(*n* = 367)	(*n* = 1835)			
Any APR test	321 (87%)	1482 (81%)	6%	1.68 (1.20–2.35)	0.001
Platelet	308 (84%)	1324 (72%)	12%	2.05 (1.52–2.77)	<0.001
Albumin	299 (81%)	1359 (74%)	7%	1.55 (1.17–2.07)	0.002
IM (ESR / CRP)	189 (52%)	743 (40%)	12%	1.59 (1.26–2.00)	<0.001
Ferritin	72 (20%)	252 (14%)	6%	1.54 (1.15–2.06)	0.005
WBC	308 (84%)	1322 (72%)	12%	2.07 (1.53–2.79)	<0.001
Haemoglobin	308 (84%)	1331 (73%)	11%	2.01 (1.49–2.72)	<0.001
Females with Abdominal Bloating (*n* = 4686)					
	(*n* = 781)	(*n* = 3905)			
Any APR test	669 (86%)	3121 (80%)	6%	1.51 (1.22–1.88)	<0.001
Platelet	627 (80%)	2869 (73%)	7%	1.48 (1.22–1.79)	<0.001
Albumin	616 (79%)	2761 (71%)	8%	1.57 (1.30–1.90)	<0.001
IM (ESR / CRP)	412 (53%)	1759 (45%)	8%	1.36 (1.17–1.59)	<0.001
Ferritin	160 (20%)	739 (19%)	1%	1.11 (0.91–1.34)	0.31
WBC	630 (81%)	2871 (74%)	7%	1.51 (1.24–1.83)	<0.001
Haemoglobin	629 (81%)	2882 (74%)	7%	1.47 (1.22–1.78)	<0.001

*APR* Acute phase reactant, *IM* inflammatory marker, *ESR* erythrocyte sedimentation rate, *CRP* c-reactive protein; *P* value from likelihood-ratio test from conditional logistic regression models (separate for each test) where case status was the outcome, and each inflammatory marker test related variable was the exposure.

On examining monthly rates of blood test use over time before cancer diagnosis a diagnostic window [26] was evident, with a marked increase in use from 7 and 6 months before cancer diagnosis in patients presenting with abdominal pain and bloating, respectively. Blood test use in controls over the same period increased but to a lesser extent (Fig. 1, Supplementary Figure S2 and Supplementary Table S4). In patients with as-yet-undetected cancer, pre-diagnostic primary care blood test use increased 5–19 fold from baseline in abdominal pain patients (RR 4.56–19.42) and 4–8 fold in abdominal bloating (RR 3.85–8.46), compared to a 2–4 fold increase in controls (RR 2.13–4.04) (Fig. 1 and Supplementary Fig. S2). The most frequently requested blood test was the full blood count (FBC), with FBC use increasing from a baseline monthly rate of 58 tests per 1000 patients to 384 per 1000 patients in male abdominal pain cases, and from 83 tests to 419 tests per 1000 patients in male abdominal bloating cases (Supplementary Fig. S2).

### Abnormal test results

#### Proportion of patients with abnormal results

Among cancer patients presenting to their GP with abdominal pain or bloating, one in three males (38–39%) and one in four females (23–28%) tested were anaemic at any point in the year pre-diagnosis (vs 22% and 11–13% of controls, respectively). Two in three symptomatic cancer patients tested had at least one abnormal APR result (59–65% vs 35–43% of controls, respectively), with similar findings in both sexes (Table 2). The most common blood test abnormality in patients with as-yet-undetected cancer was a raised inflammatory marker, with 74–79% of tested patients having at least one abnormal erythrocyte sedimentation rate (ESR) or C-reactive protein (CRP) result pre-diagnosis (Table 2). In symptomatic patients with cancer, the monthly percentage of patients with at least one blood test abnormality increased progressively from 7 months pre-diagnosis, from a baseline of <3% in the 24th month pre-diagnosis up to 19% in the month immediately preceding diagnosis. The percentage of symptomatic controls who had blood test abnormalities remained consistently ≤5% per month across all tests throughout this period (Supplementary Fig. S3 and S4). Examination of the cumulative percentage of patients with a blood test abnormality starting from 24 months pre-diagnosis showed steady linear increases over time for all tests in controls, compared to marked increases in the proportion of cases with abnormalities from 6-months before cancer diagnosis, with a clear deviation from the linear trend (Supplementary Fig. S3 and S4).

**Table 2:** Association between cancer diagnosis and abnormal blood test results in the year preceding diagnosis/index date in patients presenting with abdominal pain(*n* = 56562) or bloating (*n* = 6888)

Blood test abnormality	Cancer Patients *n* (% out of all tested patients)	Controls *n* (% out of all tested patients)	Absolute difference	Odds Ratio (95%CI)	*P* value
Males with Abdominal Pain (*n* = 26232)					
	(*n* = 4372)	(*n* = 21860)			
Any abnormal APR test	2412 (64%)	5777 (35%)	29%	3.30 (3.05–3.56)	<0.001
Raised platelet count	526 (15%)	653 (5%)	10%	3.59 (3.15–4.10)	<0.001
Low albumin	661 (19%)	1037 (7%)	12%	3.31 (2.95–3.72)	<0.001
Raised IM (ESR / CRP)	1845 (78%)	4260 (55%)	23%	3.16 (2.78–3.60)	<0.001
Raised ferritin	167 (19%)	374 (15%)	4%	1.50 (1.06–2.12)	0.02
Low Ferritin	214 (24%)	389 (15%)	9%	1.70 (1.23–2.34)	0.001
Raised total WBC count	687 (19%)	1397 (10%)	9%	2.22 (2.00–2.47)	<0.001
Anaemia	1404 (39%)	3065 (22%)	17%	2.70 (2.47–2.94)	<0.001
Females with Abdominal Pain (*n* = 30330)					
	(*n* = 5055)	(*n* = 25275)			
Any abnormal APR test	2689 (65%)	7902 (43%)	22%	2.42 (2.25–2.61)	<0.001
Raised platelet count	931 (24%)	1360 (8%)	16%	3.49 (3.16–3.85)	<0.001
Low albumin	685 (18%)	1068 (7%)	11%	3.28 (2.92–3.68)	<0.001
Raised IM (ESR / CRP)	2078 (79%)	6149 (64%)	15%	2.18 (1.94 – 2.46)	<0.001
Raised ferritin	110 (10%)	189 (5%)	5%	1.96 (1.30–2.95)	0.001
Low Ferritin	262 (25%)	665 (19%)	6%	1.48 (1.14–1.93)	0.004
Raised total WBC count	687 (17%)	1403 (8%)	9%	2.25 (2.03–2.50)	<0.001
Anaemia	1091 (28%)	2102 (13%)	15%	2.82 (2.57–3.09)	<0.001
Males with Abdominal Bloating (*n* = 2202)					
	(*n* = 367)	(*n* = 1835)			
Any abnormal APR test	188 (59%)	526 (35%)	24%	2.55 (1.98–3.28)	<0.001
Raised platelet count	35 (11%)	43 (3%)	8%	3.69 (2.25 – 6.04)	<0.001
Low albumin	57 (19%)	110 (8%)	11%	2.50 (1.72 – 3.53)	<0.001
Raised IM (ESR / CRP)	139 (74%)	401 (55%)	19%	2.46 (1.66 – 3.64)	<0.001
Raised ferritin	12 (17%)	25 (10%)	7%	1.06 (0.32 – 3.55)	0.92
Low Ferritin	13 (19%)	39 (16%)	3%	1.42 (0.55 – 3.70)	0.47
Raised total WBC count	50 (16%)	106 (8%)	8%	2.31 (1.57 – 3.38)	<0.001
Anaemia	116 (38%)	299 (22%)	16%	2.43 (1.82 – 3.25)	<0.001
Females with Abdominal Bloating (*n* = 4686)					
	(*n* = 781)	(*n* = 3905)			
Any abnormal APR test	423 (63%)	1275 (41%)	22%	2.51 (2.10–3.01)	<0.001
Raised platelet count	151 (24%)	189 (7%)	17%	4.78 (3.68–6.21)	<0.001
Low albumin	121 (20%)	128 (5%)	15%	6.23 (4.51–8.62)	<0.001
Raised IM (ESR / CRP)	318 (77%)	997 (57%)	20%	2.57 (1.94–3.40)	<0.001
Raised ferritin	13 (8%)	19 (3%)	5%	2.53 (0.68–9.35)	0.16
Low Ferritin	39 (24%)	144 (20%)	4%	1.08 (0.57–2.04)	0.81
Raised total WBC count	89 (14%)	191 (7%)	7%	2.20 (1.67–2.91)	<0.001
Anaemia	147 (23%)	303 (11%)	12%	2.80 (2.21– 3.56)	<0.001

*APR* Acute phase reactant, *IM* inflammatory marker, *ESR* erythrocyte sedimentation rate, *CRP* c-reactive protein; *P* value from likelihood-ratio test from conditional logistic regression models (separate for each test) where case status was the outcome, and each inflammatory marker test related variable was the exposure.

#### Association between blood test abnormalities and cancer diagnosis

Among abdominal pain patients, abnormalities in each of the blood tests examined were more likely in patients with cancer than those without. Cancer patients had 2 to 3-fold increased odds of having any abnormal APR or anaemia result in the year before diagnosis compared to controls (OR 2.42–3.30). Across both sexes, the strongest associations between individual blood tests and cancer were for raised platelets and low albumin, with these abnormalities being 3 to 4-fold more likely in abdominal pain patients with cancer than controls (OR 3.28–3.59). Similar results were observed in the abdominal bloating group, with abnormal results in each of the blood tests examined more likely in patients with cancer than those without, except for ferritin where no difference was found. The strongest associations were seen in women with bloating, where cancer patients had 5-fold higher odds of having raised platelets (OR 4.78, 95%CI 3.68–6.21, *P* < 0.001) and 6-fold higher odds of having a low albumin (OR 6.23, 95%CI 4.51–8.62, *p* < 0.001) than controls (Table 2).

#### Trends over time in blood test abnormalities before cancer diagnosis

**Fig. 2:**
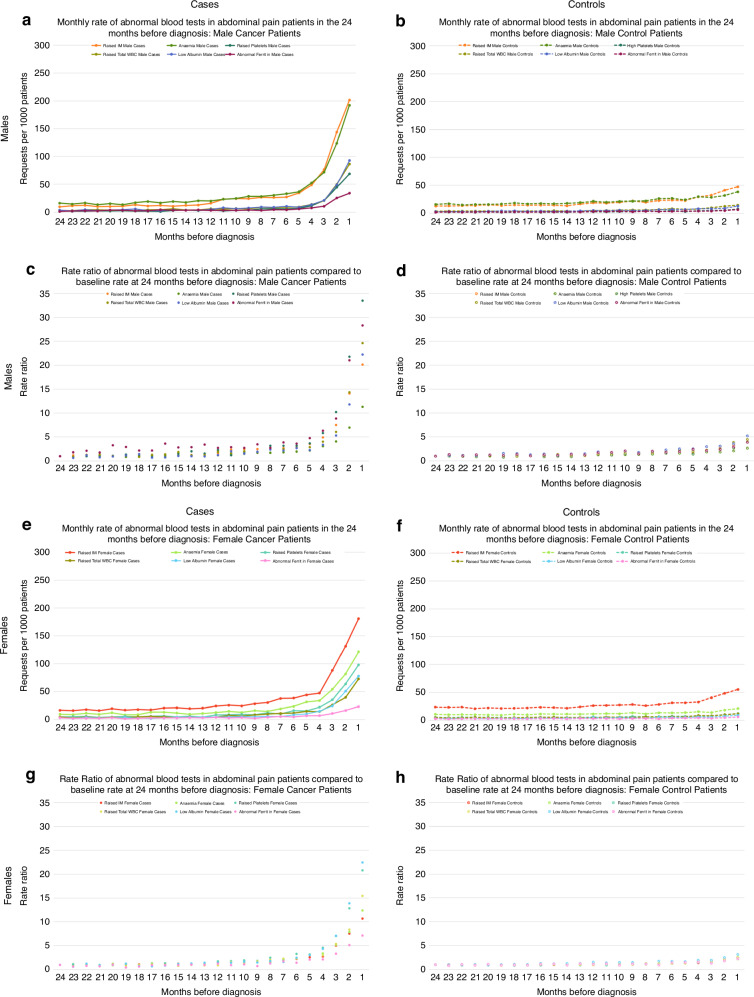
Monthly rates and rate ratios for abnormal GP blood tests in cases and controls presenting with abdominal pain in the 24 months before diagnosis/index date. **a**, **b** Display request rates and **c**, **d** display rate ratios compared to baseline rate at 24 months before diagnosis/index date in males; **e**, **f** display request rates and **g**, **h** display rate ratios compared to baseline rate at 24 months before diagnosis/index date in females. IM inflammatory marker, WBC white blood cell count.

**Fig. 3:**
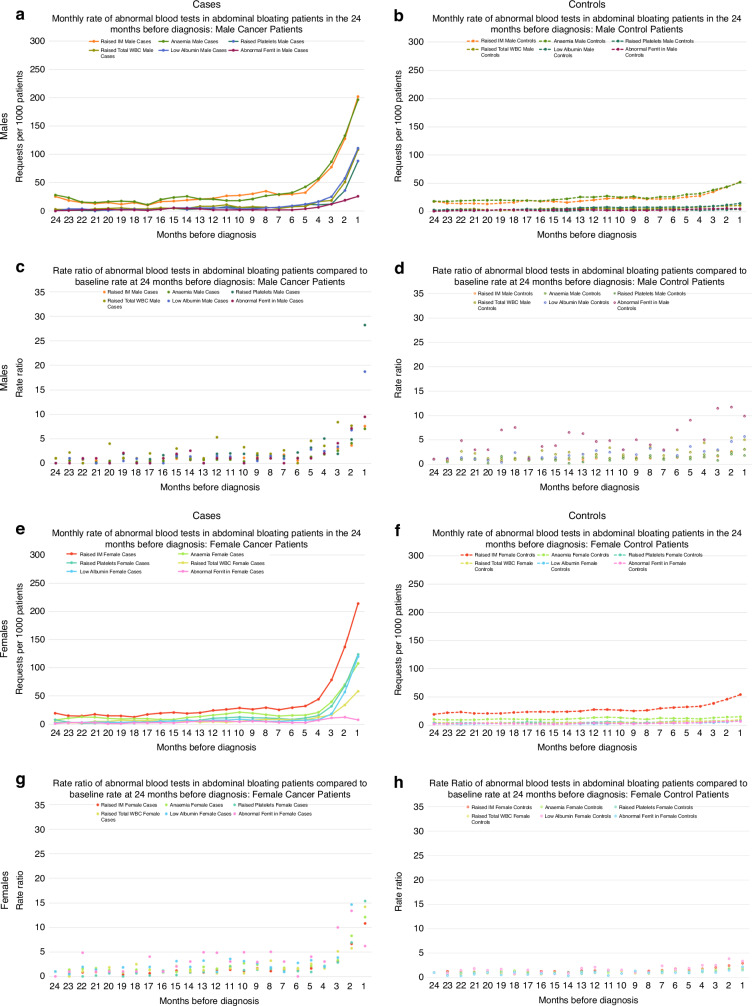
Monthly rates and rate ratios for abnormal GP blood tests in cases and controls presenting with abdominal bloating in the 24 months before diagnosis/index date. **a**, **b** Display request rates (3 month moving average) and **c**, **d** display rate ratios compared to baseline rate at 24 months before diagnosis/index date in males; **e**, **f** display request rates (3 month moving average) and **g**, **h** display rate ratios compared to baseline rate at 24 months before diagnosis/index date in females. IM inflammatory marker, WBC white blood cell count.

Examination of trends over time in blood test abnormalities among patients with abdominal pain or bloating subsequently diagnosed with cancer showed progressive increases in the rate of abnormalities from baseline starting from 7 months before diagnosis (Fig. 1 and Supplementary Table S4). Rates of blood test abnormalities in symptomatic controls also increased during this period but to a much lesser extent, with the baseline monthly rate of <50 abnormalities/1000 patients increasing to 260–316/1000 patients in cancer patients and to 67–73/1000 patients in controls in the month immediately preceding diagnosis (Fig. 1). In cancer patients, similar trends were seen across all test types examined, except for ferritin abnormalities in female cases with bloating, where no increase was seen pre-diagnosis. In symptomatic controls smaller increases were seen in abnormalities for each test, with the biggest increases seen in inflammatory marker abnormalities (in both sexes) and anaemia rates (in males) (Figs. 2 and 3).

Among cancer patients presenting with either abdominal symptom, the frequency of abnormalities differed by sex and test type. In males, raised inflammatory markers and anaemia were the most common abnormalities (Table 2), which markedly increased from 5–6 months pre-diagnosis. In females, raised inflammatory markers were also most common, with comparable increases to those seen in males from 5 months pre-diagnosis, but anaemia was less common than in males (Figs. 2 and 3). The biggest increases in abnormalities from the baseline rate were for raised platelets in males with abdominal pain (increased 33-fold), raised total WBC count in males with abdominal bloating (increased 37-fold) and low albumin in females with either symptom (increased 22-fold in abdominal pain and 41-fold in abdominal bloating patients). In symptomatic controls, monthly rate ratios for abnormalities compared to baseline remained ≤5 throughout the same period (Figs. 2 and 3).

## Discussion

Primary care blood test use increases from 7 months before cancer diagnosis in patients presenting with abdominal pain or bloating. During this time repeat primary care consultations for these symptoms are up to three-fold more likely in cancer patients, particularly for abdominal pain. This period represents a ‘diagnostic time window’ during which potential opportunities exist for earlier cancer diagnosis if these patients can be identified. Patients presenting with abdominal symptoms who do not have cancer experience much smaller increases in primary care activity. This suggests that GPs are already partially differentiating a proportion of symptomatic patients with as-yet-undetected cancer for blood testing from those without. Abnormal results in six different blood tests increase in frequency from 7 months before cancer diagnosis in patients with abdominal symptoms, suggesting they could be early signals of undetected cancer, particularly raised platelets, raised inflammatory markers and low albumin. Much smaller increases in these blood test abnormalities are seen in symptomatic patients who were not diagnosed with cancer in this time period. They therefore could potentially be used to discriminate between the two groups of patients to prioritise individuals presenting with abdominal symptoms in need of further cancer investigation or referral, if supported by advances in diagnostic technologies.

### Comparison with the literature

The ‘diagnostic time window’ is a concept which uses the time point when healthcare use first starts to increase from baseline before diagnosis to define the period when opportunities for potential earlier diagnosis exists [26, 37]. Previous proof-of-concept studies of Hodgkin lymphoma, lung, colorectal, breast, pancreatic and prostate cancer patients have shown that primary care consultations [26, 27, 38, 39], imaging requests [27, 39] and prescriptions [26, 40, 41] increase up to a year before cancer diagnosis. This indicates that some patients with as-yet-undetected cancer are presenting with symptoms and experience increased diagnostic activity in primary care an appreciable length of time before their cancer is detected. Blood test requests have also been shown to increase pre-diagnosis, with haemoglobin requests increasing up to 17 months before colorectal cancer diagnosis [26], APR and FBC requests increasing from 12 months before Hodgkin lymphoma diagnosis [24], and blood glucose and haemoglobin requests increasing from 12 months before pancreatic cancer diagnosis [27]. This demonstrates that primary care blood test requests can also be used to estimate diagnostic windows and that, for some, cancer diagnosis could potentially be expedited by several months if patients undergoing testing *with as-yet-undetected cancer* can be identified and differentiated from those without. Existing studies on diagnostic activity did not take symptoms into account and focus on a single cancer site. Our findings are consistent with previous findings of diagnostic windows existing in primary care before cancer diagnosis, but extends previous findings by examining this phenomenon in a symptomatic population (of cases and controls). It confirms that in patients presenting with the same abdominal symptoms, primary care blood test use is more likely in patients with as-yet-undetected cancer and these activities increase several months before cancer diagnosis of any site with much smaller increases occurring in symptomatic controls. This finding concords with a previous study indicating that GPs preferentially select patients at higher risk when deciding on urgent referrals for suspected cancer [42]. However, we also observe that this selection is not adequate/sufficient to expedite the diagnosis, as diagnostic windows of substantial length are still observed.

A small number of studies have examined the timing of blood test abnormalities before cancer diagnosis. Rates of anaemia, a well recognised feature of many cancer types [43], have been shown to increase up to 9 months before colorectal cancer diagnosis [35] and abnormalities in certain APR tests increase up to a year before Hodgkin lymphoma diagnosis [24] and up to 8 months before CRC diagnosis [35]. Our study builds on these findings by showing that, in the diagnostically challenging cohort of patients who present to general practice with abdominal pain or bloating, abnormalities in six commonly used blood tests increase several months before cancer diagnosis of any site. Additionally, by comparing with symptomatic controls we show that these APR blood test results could help to differentiate symptomatic patients with and without as-yet-undetected cancer, if supported by advances in diagnostic technologies to help identify these patients and prompt earlier cancer investigation.

### Strengths and limitations

A main strength of this study is that it is set in primary care where most symptomatic cancer patients first present [44] and uses a large sample of CPRD data, which is representative of the UK population [32]. In common with all routinely collected coded data, CPRD will not capture symptomatic presentations solely recorded as free text in the medical record, therefore the results relate to patients who have presented to primary care with the symptom and where the clinician considered it important enough to be coded. Wide variability exists in coding practices between GPs [45], but any effects of clinician recording bias of symptoms are reduced as controls were selected from the same coded population as cases. Information on the severity of symptoms was not available. This should be examined in future studies, as more severe symptoms may confer greater risk of as-yet-undetected cancer. Primary care blood test results are electronically transmitted to the medical record from laboratories ensuring their accuracy and completeness, and cancer diagnoses were identified using linkage to the English national cancer registry, which is the gold-standard for cancer identification [46]. Cancer registration data were available in the linked dataset up to October 2017. It is possible that patient and clinician behaviours including propensity/access to consult with symptoms and use of blood test may change over time, particularly post COVID-19. Future research to examine this should be conducted once linked cancer registry data post 2020 becomes available.

Another strength of this study was the use of statistical techniques to accurately estimate the length of the diagnostic window [34, 37] by modeling the most likely inflection point for increased diagnostic activity. This addresses concerns raised about some previous methodologies for estimating diagnostic window length [37]. The novel use of a symptomatic comparator group also enabled us to assess the discriminatory value of different tests for identifying cancer patients from all patients with the recorded symptom. Previous diagnostic windows studies have either not used comparator groups or the comparator group was not ‘matched’ for recorded symptomatic presentation, which allows estimation of the diagnostic window but not appreciation of distinguishing features between symptomatic cases and controls [37].

It should be noted that the analyses in our study examine population level trends and these will not necessarily be reflected in individual patients presenting with these symptoms in primary care. Further research is therefore needed to examine the predictive value of the identified blood tests abnormalities for identifying as-yet-undetected cancer at the individual level, to determine if they can inform decisions on cancer investigation and referral. Another limitation is that the sole outcome of interest was any cancer diagnosis in patients presenting with abdominal symptoms. This outcome was selected as non-specific abdominal symptoms are features of many cancer sites [21, 22], however it does not allow evaluation of potentially distinct patterns of blood test abnormalities for different cancer sites. Further work examining pre-diagnostic blood trends by cancer site and stage could support prioritisation of malignancies for further investigation in patients with non-specific symptoms. We have not adjusted for comorbidity; it is possible that cancer patients have higher rates of blood testing and increased healthcare use compared to controls due to greater comorbidity burden in cases, and if so this factor would artefactually increase the observed testing differences. Future studies may adjust for comorbidity. Further, we did not adjust for general practice; different practices may have different propensity to test patients, but this may only lead to random misestimation of true differences in testing activity between cases and controls (e.g. if cases are over-represented in higher than average testing propensity practices or vice versa, due to chance).

### Implications

The findings of this study have important clinical and research implications. In some patients with abdominal pain or bloating who have ‘as-yet-undetected’ cancer, primary care activity starts to increase from 7 months before diagnosis. During this period, repeat consultations for the same abdominal symptom are common and are up to three-fold more likely to occur than in patients who were not diagnosed with cancer in this time period. This indicates that many cancer patients are repeatedly presenting in primary care (and being investigated with blood tests) and opportunities may therefore exist to bring their diagnosis forward by several months if these patients can be identified. Repeat consultations are a well-recognised feature of the diagnostic pathway for many cancer patients, especially those presenting with non-specific symptoms [10, 16, 47]. Although repeat consultations for abdominal pain and bloating on their own have relatively low predictive value for cancer [48, 49], these findings provide further evidence that these events should prompt consideration of possible cancer by GPs.

The increase in blood test use seen in cancer patients with abdominal symptoms was much larger than in patients who did not have cancer. This indicates that a degree of triage is already being done by GPs in patients who present with these symptoms to select those at higher risk of cancer for blood test investigations [42, 50]. This selection could be based on ‘gut feelings’ or other differences between cases and controls related to their clinical presentation or patient features not captured in coded symptoms [51–53]. Further diagnostic advances are needed to support GPs in identifying which of these tested patients are at highest risk of as-yet-undetected cancer and prompt earlier cancer investigation/referral.

Six blood test abnormalities in this study were found to increase in patients with as-yet-undetected cancer during the identified diagnostic window and are likely early signals of cancer. This is particularly relevant as these increases in abnormalities were much less frequent in symptomatic patients who were not diagnosed with cancer in this time period and they therefore have potential value in differentiating tested symptomatic patients in need of further cancer assessment. Further studies are needed to better understand why, for some symptomatic patients with blood test abnormalities, cancer diagnosis occurred after several months. As primary care blood test abnormalities are common and not specific to cancer, each result in isolation will likely have low predictive value for cancer. Nonetheless, when combined with other results and accompanying pre-diagnostic events recorded in the electronic health record, such as symptoms, prescriptions and consultations, they could help identify which patients with abdominal symptoms should be prioritised for cancer investigation or referral [31, 54, 55]. Future research should examine the predictive value of combinations of the blood test abnormalities identified in this study alongside other concurrent features for as-yet-undetected cancer in patients with abdominal pain or bloating.

## Conclusion

In conclusion, in patients who present to primary care with abdominal pain or bloating and are subsequently diagnosed with cancer there is potential for expediting diagnosis in some patients by up to seven months. Abnormal APR blood test results are more common in symptomatic patients with as-yet-undetected cancer than those without and are early signals of cancer. Future work should examine the predictive value of these blood test abnormalities alongside other features in primary care records to develop diagnostic tools to help differentiate and triage patients with vague abdominal symptoms presenting to primary care who should be prioritised for urgent cancer investigation.

## Supplementary information

Supplementary data

STROBE-checklist

## Data Availability

This study is based in part on data from the Clinical Practice Research Datalink obtained under licence from the UK Medicines and Healthcare products Regulatory Agency. The data is provided by patients and collected by the NHS as part of their care and support. Due to privacy laws and the data user agreement between UCL and CPRD, researchers are not authorised to share individual patient data. Access to CPRD data, including UK Primary Care Data, and linked data such as Hospital Episode Statistics, is subject to protocol approval via CPRD’s Research Data Governance (RDG) Process, see https://cprd.com/data-access for further details. As CPRD uses de-identified patient data individual patient consent is not required, however, patients can opt out of their patient information being shared for research.

## Ethics approval

The protocol for this project was approved by the Independent Scientific Advisory Committee (ISAC) for MHRA Database Research (protocol number:18_299R). Generic ethical approval for observational studies conducted using anonymised CPRD data with approval from ISAC has been granted from a National Research Ethics Service Committee (NRESC). The study was performed in accordance with the Declaration of Helsinki. All methods were performed in accordance with the relevant guidelines and regulations.
