# Supplementary material for: A UK population-based case-control study of blood tests before cancer diagnosis in patients with non-specific abdominal symptoms
Source: Br J Cancer. 2025 Jan 11;132(5):450–61. doi: 10.1038/s41416-024-02936-9 (PMC11876449; doi:10.1038/s41416-024-02936-9)
Supplement: Supplementary file 1 — Supplementary data [file 41416_2024_2936_MOESM1_ESM.docx]

## Supplementary Data

##### Supplementary Table S1: Read codes for abdominal pain and bloating

| Read Code | Term |
| --- | --- |
| 1969.00 | Abdominal pain |
| 1972.00 | Epigastric pain |
| 197..12 | Iliac fossa pain |
| R090500 | [D]Epigastric pain |
| R090700 | [D]Hypochondrial pain |
| 1975.00 | Left flank pain |
| R090400 | [D]Abdominal cramps |
| R090F00 | [D]Acute abdomen |
| 1977.00 | Right iliac fossa pain |
| 1829.00 | Retrosternal pain |
| R090200 | [D]Colic NOS |
| R090.00 | [D]Abdominal pain |
| 196..11 | Abdominal pain type |
| R090100 | [D]Abdominal colic |
| R090E00 | [D]Recurrent acute abdominal pain |
| 1968.00 | Abdominal discomfort |
| 1978.00 | Left iliac fossa pain |
| 1976.00 | Right flank pain |
| R090z00 | [D]Abdominal pain NOS |
| 197B.00 | Upper abdominal pain |
| 1971.00 | Central abdominal pain |
| R090600 | [D]Umbilical pain |
| 1963.00 | Non-colicky abdominal pain |
| 25C..15 | O/E - abdomen tender |
| 2I18.12 | O/E - tenderness |
| 1A53.12 | C/O - lumbar pain |
| 197..13 | Site of abdominal pain |
| 197..14 | Subcostal pain |
| 196..12 | Type of GIT pain - symptom |
| 1979.00 | Suprapubic pain |
| 197..11 | Flank pain |
| R090J00 | [D]Right upper quadrant pain |
| 1962.00 | Colicky abdominal pain |
| R090K00 | [D]Left upper quadrant pain |
| R090H00 | [D]Upper abdominal pain |
| R090L00 | [D]Left lower quadrant pain |
| 197D.00 | Right upper quadrant pain |
| 197A.11 | General abdominal pain-symptom |
| 25C8.00 | O/E - abd. pain - R.iliac |
| 25C2.00 | O/E - abd.pain-R.hypochondrium |
| 2I18100 | Tenderness of epigastrium |
| 25CZ.00 | O/E -abd.pain on palpation NOS |
| 196..00 | Type of GIT pain |
| 25C..00 | O/E - abdo. pain on palpation |
| 2I18.00 | O/E - tenderness/pain |
| R090C00 | [D]Loin pain |
| R090000 | [D]Abdominal tenderness |
| R090900 | [D]Pain in right iliac fossa |
| R090A00 | [D]Pain in left iliac fossa |
| 25C..12 | O/E - iliac pain on palpation |
| 1DC5.00 | Griping pain |
| 25E..00 | O/E - rebound tenderness |
| 25C..14 | O/E - umbilical pain on palp. |
| 25C..13 | O/E - lumbar pain on palpation |
| 25C3.00 | O/E - abd. pain - epigastrium |
| R090N00 | [D]Nonspecific abdominal pain |
| R090M00 | [D]Right lower quadrant pain |
| R090800 | [D]Suprapubic pain |
| 25C..11 | O/E - epigastric pain on palp. |
| 25CA.00 | O/E - abd. pain - L.iliac |
| 197C.00 | Lower abdominal pain |
| 1973.00 | Left subcostal pain |
| 25D..11 | O/E - guarding of abdomen |
| 25C6.00 | O/E - abd. pain - umbilical |
| 197A.00 | Generalised abdominal pain |
| 197..00 | Site of GIT pain |
| 25C7.00 | O/E - abd. pain - L.lumbar |
| R073200 | [D]Gas pain (abdominal) |
| 1969000 | Abdominal wall pain |
| 197Z.00 | Site of GIT pain NOS |
| R090y00 | [D]Other specified abdominal pain |
| 25D..00 | O/E - guarding on palpation |
| 25D8.00 | O/E - guarding - R.iliac |
| 25C5.00 | O/E - abd. pain - R.lumbar |
| 25C4.00 | O/E - abd.pain-L.hypochondrium |
| 25D2.00 | O/E - guarding-R.hypochondrium |
| 25C9.00 | O/E - abd. pain - hypogastrium |
| 25D3.00 | O/E - guarding - epigastrium |
| 196Z.00 | Type of GIT pain NOS |
| 25F..00 | O/E - abdominal rigidity |
| Ryu1000 | [X]Pain localized to other parts of lower abdomen |
| Ryu1100 | [X]Other and unspecified abdominal pain |
| E278000 | Psychogenic pain unspecified |
| R096.00 | [D]Acute abdomen |
| 25D9.00 | O/E - guarding - hypogastrium |
| 25DA.00 | O/E - guarding - L.iliac |
| 25EZ.00 | O/E - rebound tenderness NOS |
| 25D4.00 | O/E - guarding-L.hypochondrium |
| 25D6.00 | O/E - guarding - umbilical |
| 25DZ.00 | O/E -guarding on palpation NOS |
| R090P00 | [D]Functional abdominal pain syndrome |
| 1974.00 | Right subcostal pain |
| 25F2.00 | O/E - board like abd. rigidity |
| R090311 | [D]Evening colic |
| R094.00 | [D]Abdominal rigidity |
| 19A3.00 | Abdomen feels distended |
| R073400 | [D]Bloating |
| 19B..12 | Bloating symptom |
| 19A..00 | Abdominal distension symptom |
| R073300 | [D]Abdominal distension, gaseous |
| 19A2.00 | Abdomen feels bloated |
| 19AZ.00 | Abd. distension symptom NOS |
| R073.00 | [D]Flatulence, eructation and gas pain |
| R073z00 | [D]Flatulence, eructation and gas pain NOS |
| 19B3.00 | Excessive belching |
| 19B2.00 | Excessive flatulence |
| 19B4.00 | Excessive eructation |
| R073000 | [D]Flatulence |
| R073100 | [D]Eructation |

##### Supplementary Table S2: Baseline characteristics of patients with abdominal pain and bloating who were diagnosed with cancer within 12 months compared to those without cancer diagnosis.

*matched variables; SD, standard deviation; IQR, Interquartile range; IMD, index of multiple deprivation.

|  | **Abdominal Pain Cohort** | | **Abdominal Bloating Cohort** | |
| --- | --- | --- | --- | --- |
| **Characteristics** | **Cancer Patients**  **(n=9427)** | **Controls**  **(n=47,135)** | **Cancer Patients**  **(n=1148)** | **Controls**  **(n=5,740)** |
| Male sex* | 4372 (46%) | 21860 (46%) | 367 (32%) | 1835 (32%) |
| Age at presentation* (years) |  |  |  |  |
| 30–39 | 383 (4%) | 1941 (4%) | 38 (3%) | 217 (4%) |
| 40–49 | 737 (8%) | 3761 (8%) | 92 (8%) | 525 (9%) |
| 50–59 | 1407 (15%) | 7012 (15%) | 188 (16%) | 884 (15%) |
| 60–69 | 2599 (28%) | 13343 (28%) | 312 (27%) | 1592 (28%) |
| 70–79 | 2578 (27%) | 13001 (28%) | 331 (29%) | 1608 (28%) |
| 80 and over | 1723 (18%) | 8077 (17%) | 187 (16%) | 914 (16%) |
| Mean (SD, range) | 67 (14, 30-100) | 67 (13, 30-101) | 67 (13, 30-98) | 66 (13, 30-98) |
| Median (IQR) | 68 (59-77) | 68 (59-77) | 68 (58-76) | 67 (58-76) |
| Year of Presentation* |  |  |  |  |
| 2007 | 1377 (15%) | 6890 (15%) | 113 (10%) | 565 (10%) |
| 2008 | 1422 (15%) | 7106 (15%) | 150 (13%) | 746 (13%) |
| 2009 | 1337 (14%) | 6685 (14%) | 138 (12%) | 684 (12%) |
| 2010 | 1205 (13%) | 6035 (13%) | 167 (15%) | 844 (15%) |
| 2011 | 1083 (11%) | 5403 (11%) | 146 (13%) | 732 (13%) |
| 2012 | 977 (10%) | 4886 (10%) | 135 (12%) | 682 (12%) |
| 2013 | 890 (9%) | 4448 (9%) | 116 (10%) | 570 (10%) |
| 2014 | 715 (8%) | 3585 (8%) | 110 (10%) | 552 (10%) |
| 2015 | 421 (4%) | 2097 (4%) | 73 (6%) | 364 (6%) |
| 2016 | 0 (0%) | 0 (0%) | 0 (0%) | 1 (0%) |
| Mean days follow-up  (SD, range) | 724  (38, 21-730) | 722  (44, 8-730) | 725  (40, 91-730) | 723  (46, 10-730) |
| IMD quintile |  |  |  |  |
| 1 (least deprived) | 2229 (24%) | 11429 (24%) | 295 (26%) | 1409 (25%) |
| 2 | 2139 (23%) | 10978 (23%) | 263 (23%) | 1330 (23%) |
| 3 | 1974 (21%) | 10190 (22%) | 259 (23%) | 1247 (22%) |
| 4 | 1727 (18%) | 8084 (17%) | 176 (15%) | 946 (16%) |
| 5 (most deprived) | 1353 (14%) | 6424 (14%) | 155 (14%) | 807 (14%) |

##### Supplementary Table S3: Repeat GP consultations for the same abdominal symptom before cancer diagnosis / index date

*Odds ratio and 95%CI, SD, standard deviation, P value from chi squared test

| **Male patients** | | | | | | |
| --- | --- | --- | --- | --- | --- | --- |
|  | **Abdominal Pain Cohort** | | | **Abdominal Bloating Cohort** | | |
| **GP consultations for same abdominal symptom** | **Cancer Patients**  **(n=4,372)** | **Controls**  **(n=21,860)** | **P value** | **Cancer Patients**  **(n=367)** | **Controls**  **(n=1,835)** | **P value** |
| 12 months pre-index date |  |  | <0.001 |  |  | 0.27 |
| 1 consultation | 3032 (69%) | 18814 (86%) |  | 329 (90%) | 1678 (91%) |  |
| ≥ 2 consultations | 1340 (31%) | 3046 (14%) |  | 38 (10%) | 157 (9%) |  |
| Mean (SD, range) | 1.5 (0.99, 1-11) | 1.2 (0.53, 1-13) |  | 1.1 (0.42, 1-4) | 1.1 (0.39, 1-6) |  |
| Odds of repeat presentation* | 2.80 (2.59 – 3.02) | reference | <0.001 | 1.24 (0.85 – 1.80) | reference | 0.27 |
| 6 months pre-index date |  |  | <0.001 |  |  | 0.29 |
| 0 consultations | 737 (17%) | 4321 (20%) |  | 74 (20%) | 375 (20%) |  |
| 1 consultation | 2548 (58%) | 15356 (70%) |  | 263 (72%) | 1350 (74%) |  |
| ≥ 2 consultations | 1087 (25%) | 2183 (10%) |  | 30 (8%) | 110 (6%) |  |
| Mean (SD, range) | 1.2 (1.03, 0-11) | 0.9 (0.64, 0-13) |  | 0.9 (0.59, 0-4) | 0.9 (0.54, 0-5) |  |
| Odds of repeat presentation* | 2.73 (2.54 – 2.93) | reference | <0.001 | 1.37 (0.88 – 2.13) | reference | 0.17 |
| **Female patients** | | | | | | |
|  | **Abdominal Pain Cohort** | | | **Abdominal Bloating Cohort** | | |
| **GP consultations for same abdominal symptom** | **Cancer Patients**  **(n=5,055)** | **Controls**  **(n=25,275)** | **P value** | **Cancer Patients**  **(n=781)** | **Controls**  **(n=3905)** | **P value** |
| 12 months pre-index date |  |  | <0.001 |  |  | <0.001 |
| 1 consultation | 3458 (68%) | 21493 (85%) |  | 657 (84%) | 3560 (91%) |  |
| ≥ 2 consultations | 1597 (32%) | 3782 (15%) |  | 124 (16%) | 345 (9%) |  |
| Mean (SD, range) | 1.6 (1.09, 1-12) | 1.2 (0.57, 1-12) |  | 1.2 (0.64, 1-12) | 1.1 (0.39, 1-6) |  |
| Odds of repeat presentation* | 3.07 (2.81 – 3.35) | reference | <0.001 | 2.00 (1.559 – 2.51) | reference | <0.001 |
| 6 months pre-index date |  |  | <0.001 |  |  | <0.001 |
| 0 consultations | 881 (17%) | 5438 (22%) |  | 146 (19%) | 746 (19%) |  |
| 1 consultation | 2913 (58%) | 17271 (68%) |  | 542 (69%) | 2926 (75%) |  |
| ≥ 2 consultations | 1261 (25%) | 2566 (10%) |  | 93 (12%) | 233 (6%) |  |
| Mean (SD, range) | 1.3 (1.10, 0-12) | 0.9 (0.65, 0-11) |  | 1.0 (0.65, 0-6) | 0.9 (0.52, 0-5) |  |
| Odds of repeat presentation* | 2.99 (2.75 – 3.25) | reference | <0.001 | 2.19 (1.69 – 2.86) | reference | <0.001 |

##### Supplementary Table S4: Inflection points for when the rate of blood test requests and abnormalities first start to increase from baseline before cancer diagnosis (identified from statistical modelling)

FBC, full blood count; ESR, erythrocyte sedimentation rate; CRP, c-reactive protein; LFT, liver function test

|  | **Inflection point (months before cancer diagnosis)** | | | |
| --- | --- | --- | --- | --- |
|  | **Abdominal pain patients** | | **Abdominal bloating patients** | |
|  | **Males** | **Females** | **Males** | **Females** |
| **GP consultations** | | |  |  |
| Consultations for same abdominal symptom | 5 months | 5 months | 5 months | 5 months |
| **Blood test requests** | | | | |
| Haemoglobin (part of the FBC) | 5 months | 6 months | 5 months | 4 months |
| Any acute phase reactant test | 5 months | 5 months | 5 months | 5 months |
| Ferritin | 6 months | 7 months | 6 months | 5 months |
| Inflammatory marker (ESR / CRP) | 5 months | 5 months | 6 months | 5 months |
| Albumin (part of the LFT panel) | 5 months | 5 months | 5 months | 5 months |
| **Blood test abnormalities** | | |  |  |
| Any abnormal acute phase reactant test | 5 months | 5 months | 5 months | 4 months |
| Anaemia | 5 months | 7 months | 6 months | 4 months |
| Abnormal ferritin | 5 months | 6 months | 6 months | 6 months |
| Raised inflammatory marker | 5 months | 5 months | 5 months | 4 months |
| Raised platelet count | 5 months | 5 months | 2 months | 4 months |
| Raised total white blood cell count | 5 months | 4 months | 2 months | 4 months |
| Low albumin | 4 months | 6 months | 4 months | 3 months |

##### Supplementary Figure S1: Monthly proportion of cases and controls with a GP blood test request for the 24 months before diagnosis/index date

Panels A, B, E and F: incident percentages, panels C, D, G, H: cumulative percentages over time. FBC, full blood count; LFT, liver function test; IM, inflammatory marker.

|  | Cases | Controls |
| --- | --- | --- |
| Abdominal Pain | 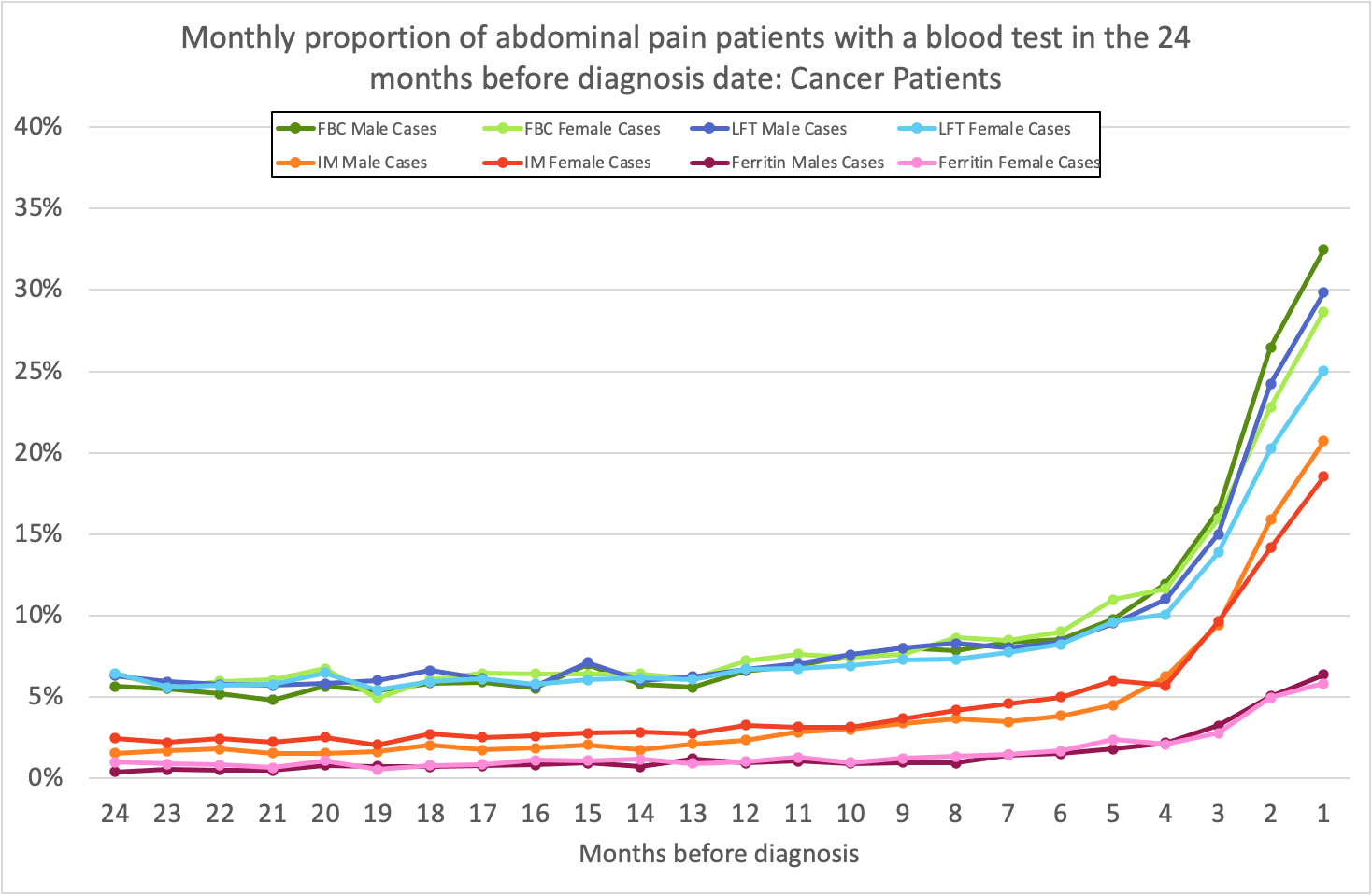  **A** | 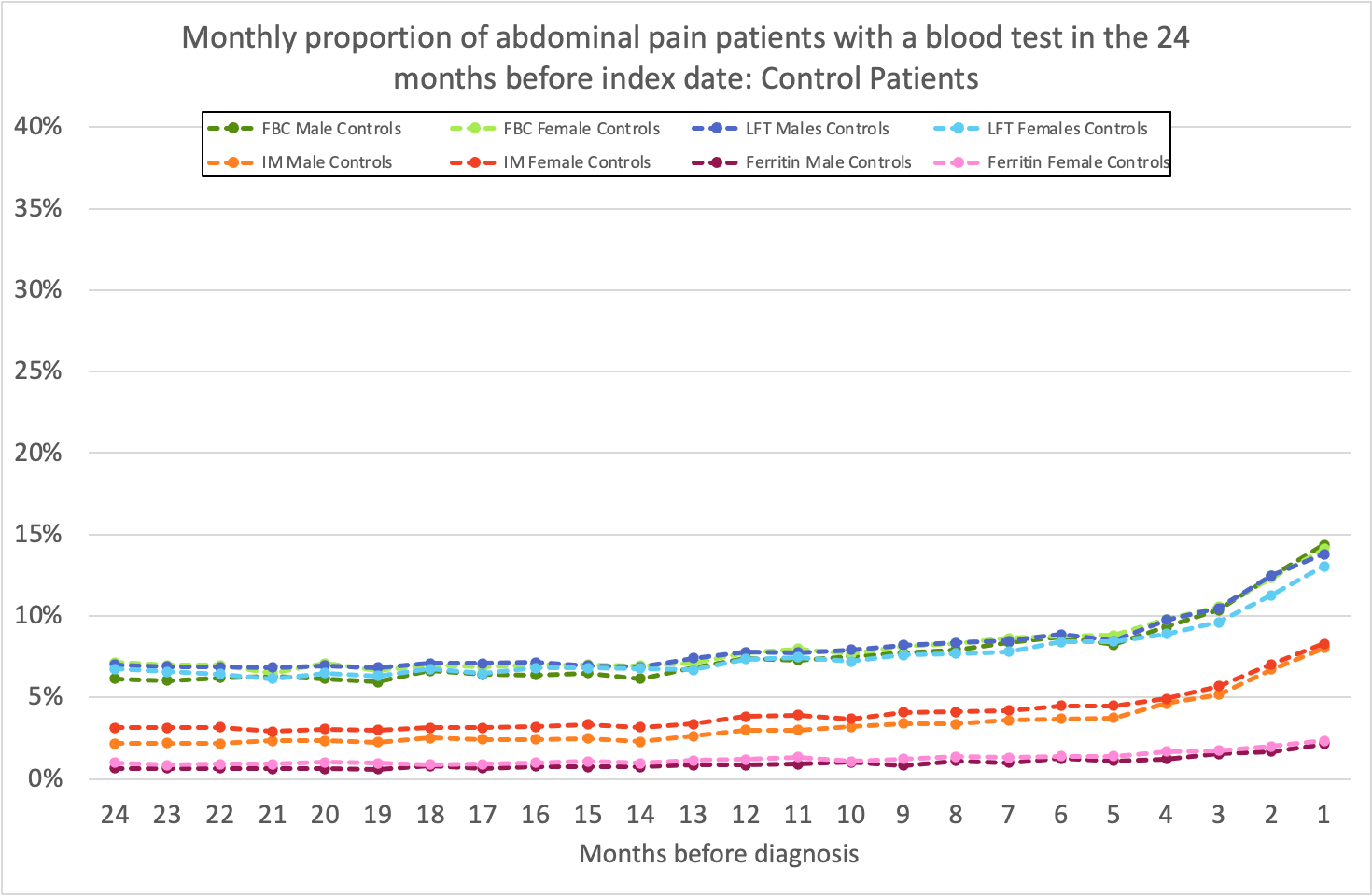  **B** |
| Abdominal Pain | 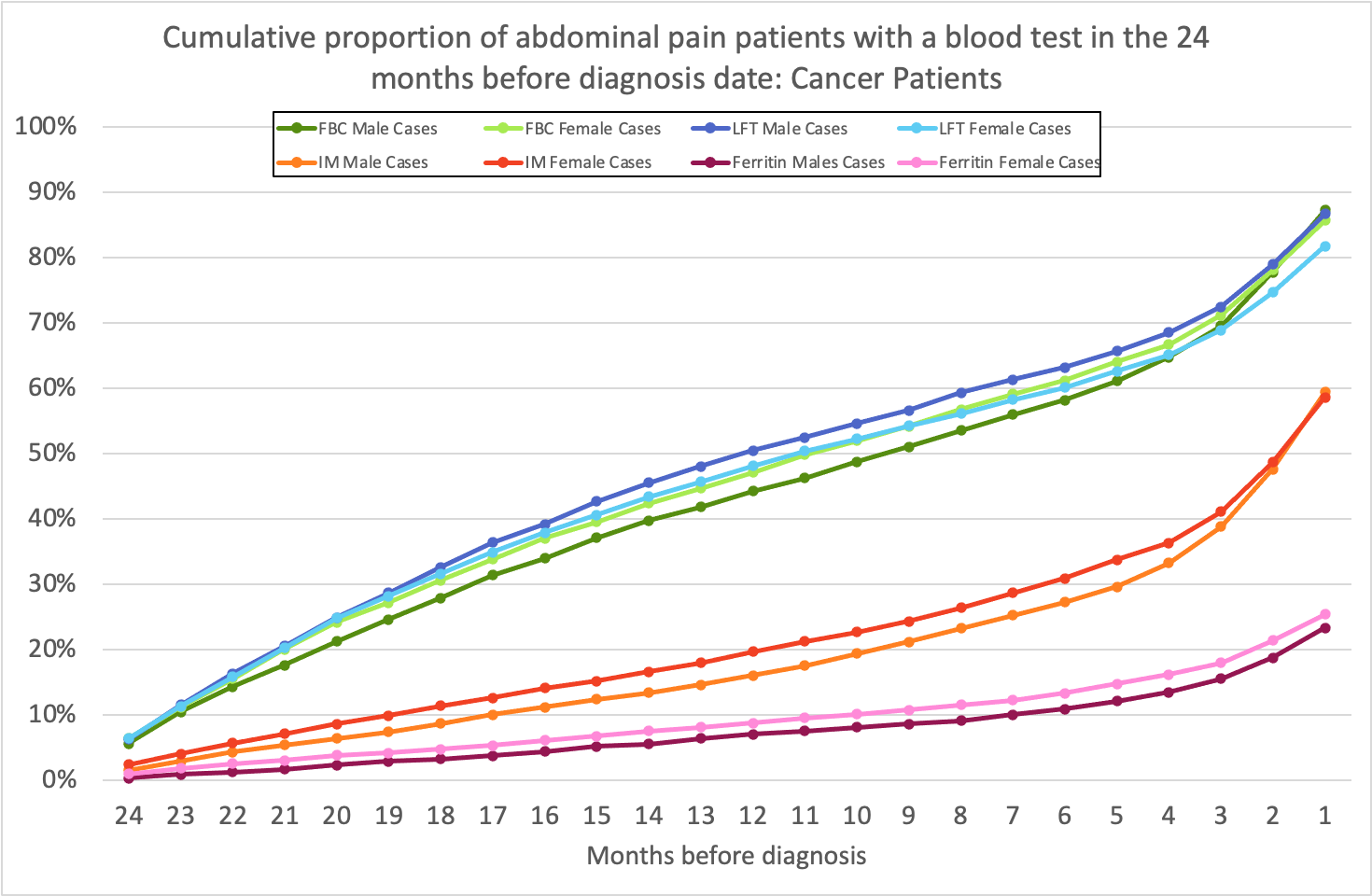  **C** | 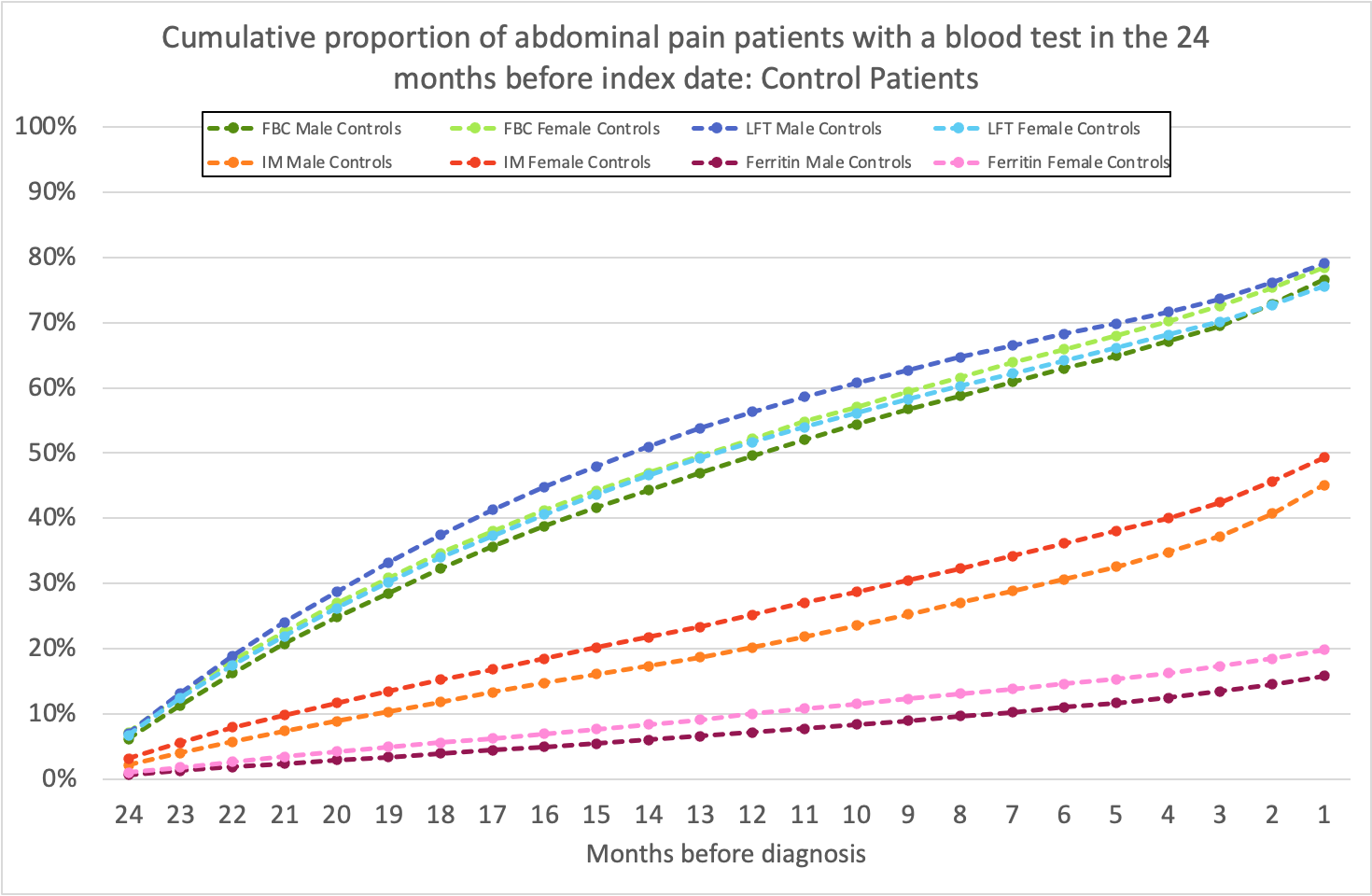  **D** |
| Abdominal Bloating |   **E** |   **F** |
| Abdominal Bloating | 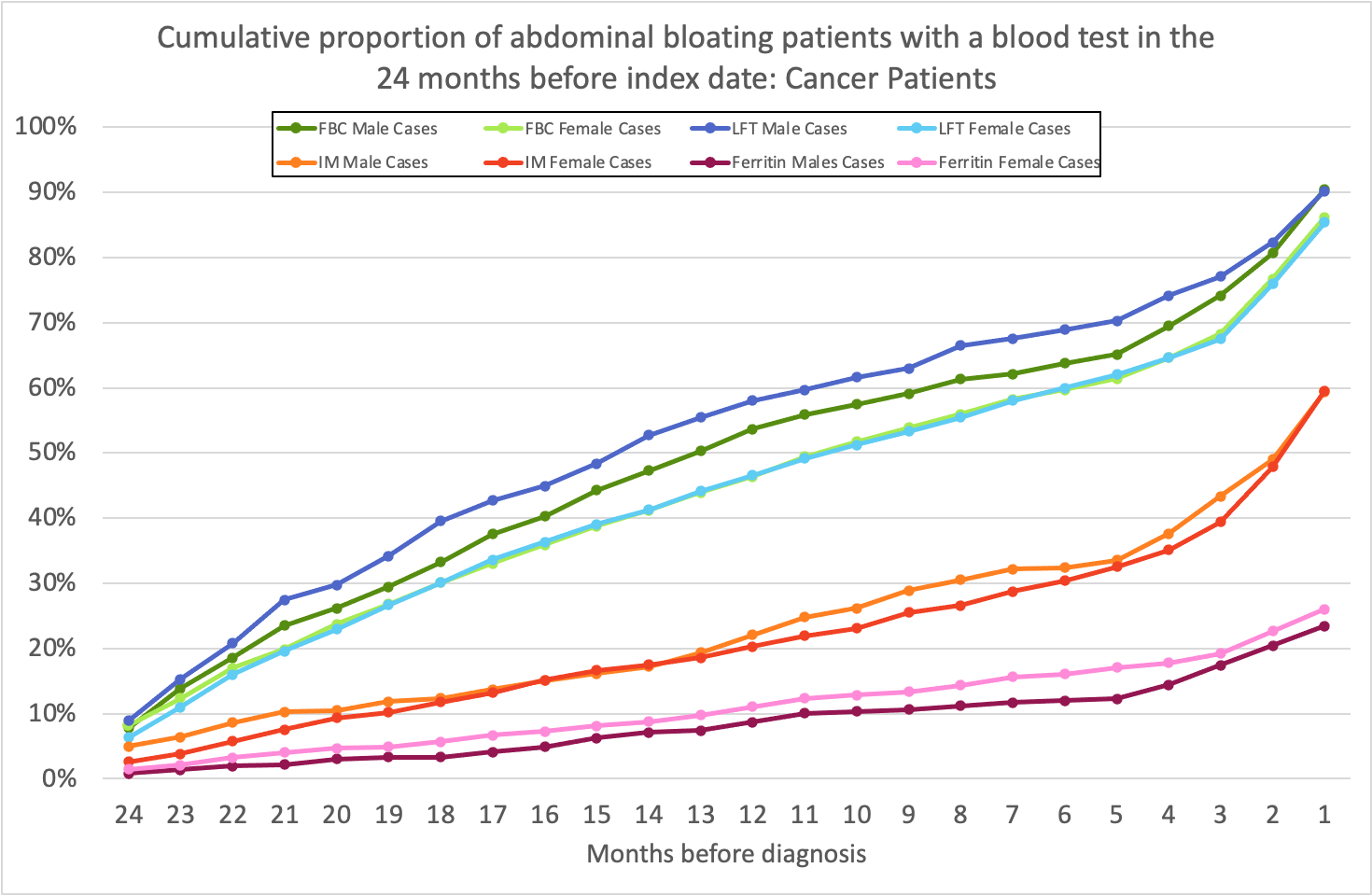  **G** | 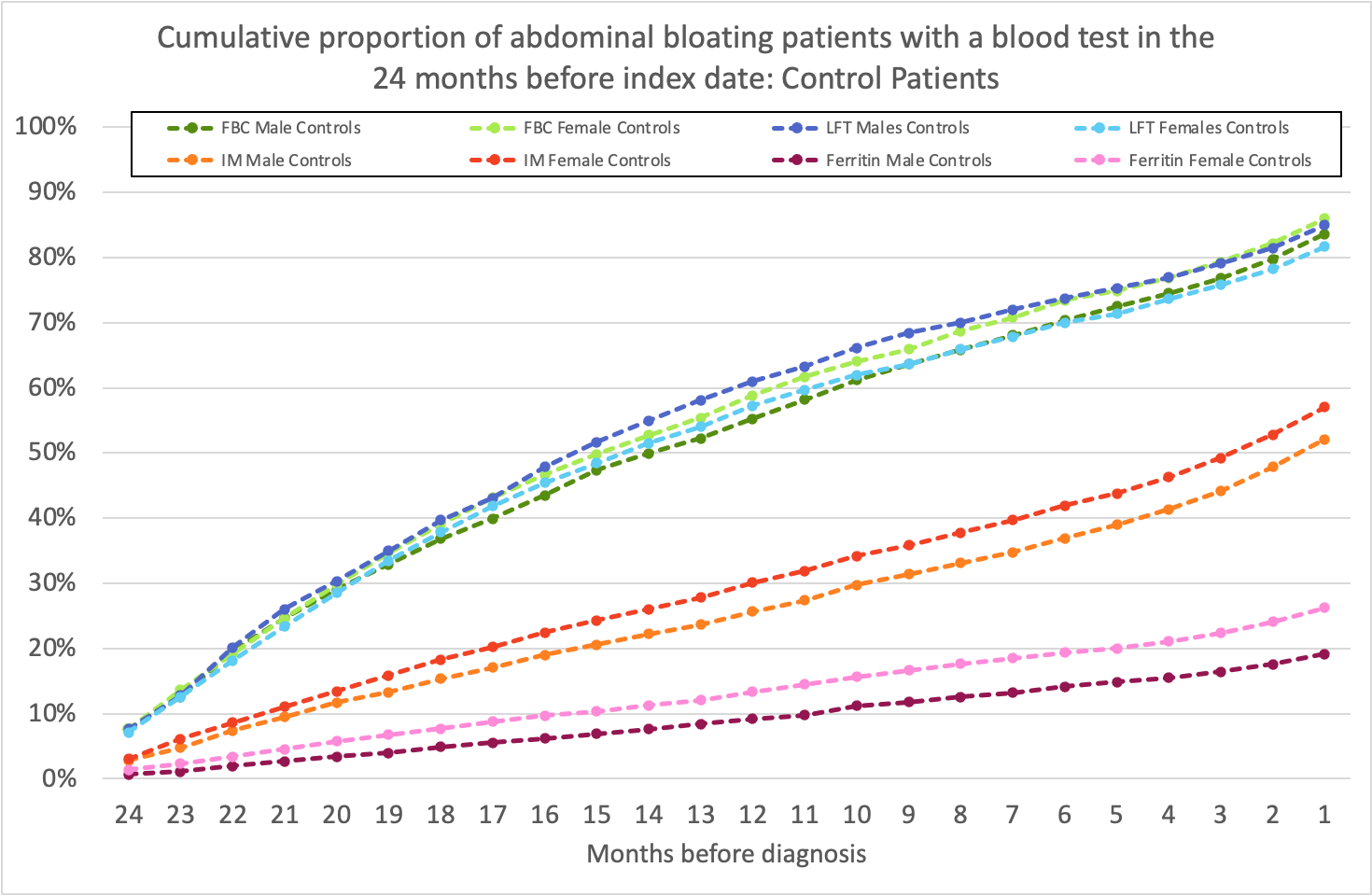  **H** |

##### Supplementary Figure S2: Monthly request rates and rate ratios for GP blood tests in cases and controls presenting with abdominal pain and bloating in the 24 months before diagnosis / index date.

Panels A, B, E, and F: request rates, panels C, D, G and H: rate ratios compared to baseline rate; FBC, full blood count; LFT, liver function test; IM, inflammatory marker.

|  | Cases | Controls |
| --- | --- | --- |
| Abdominal Pain | 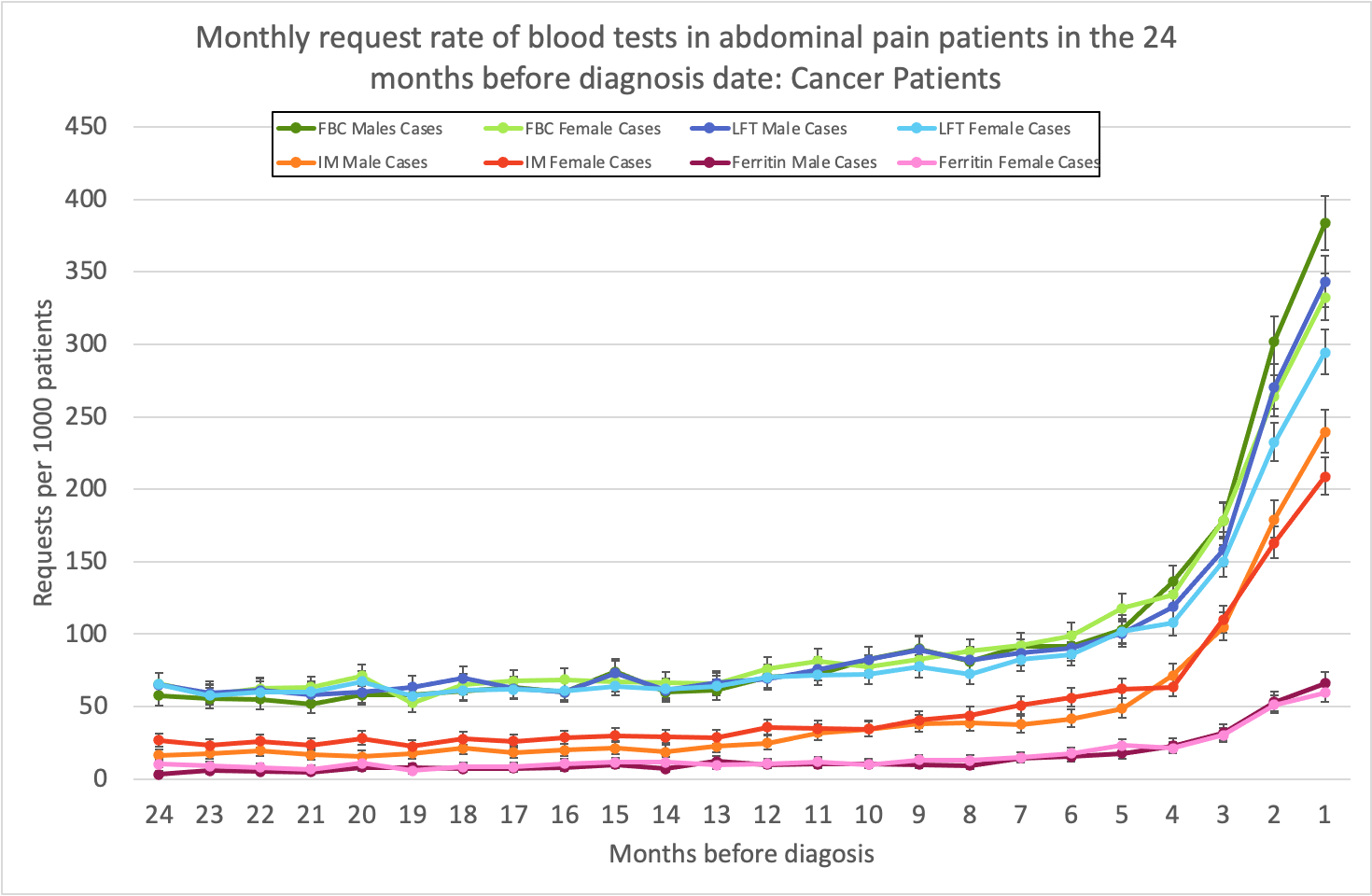  **A**  **G**  **H** | 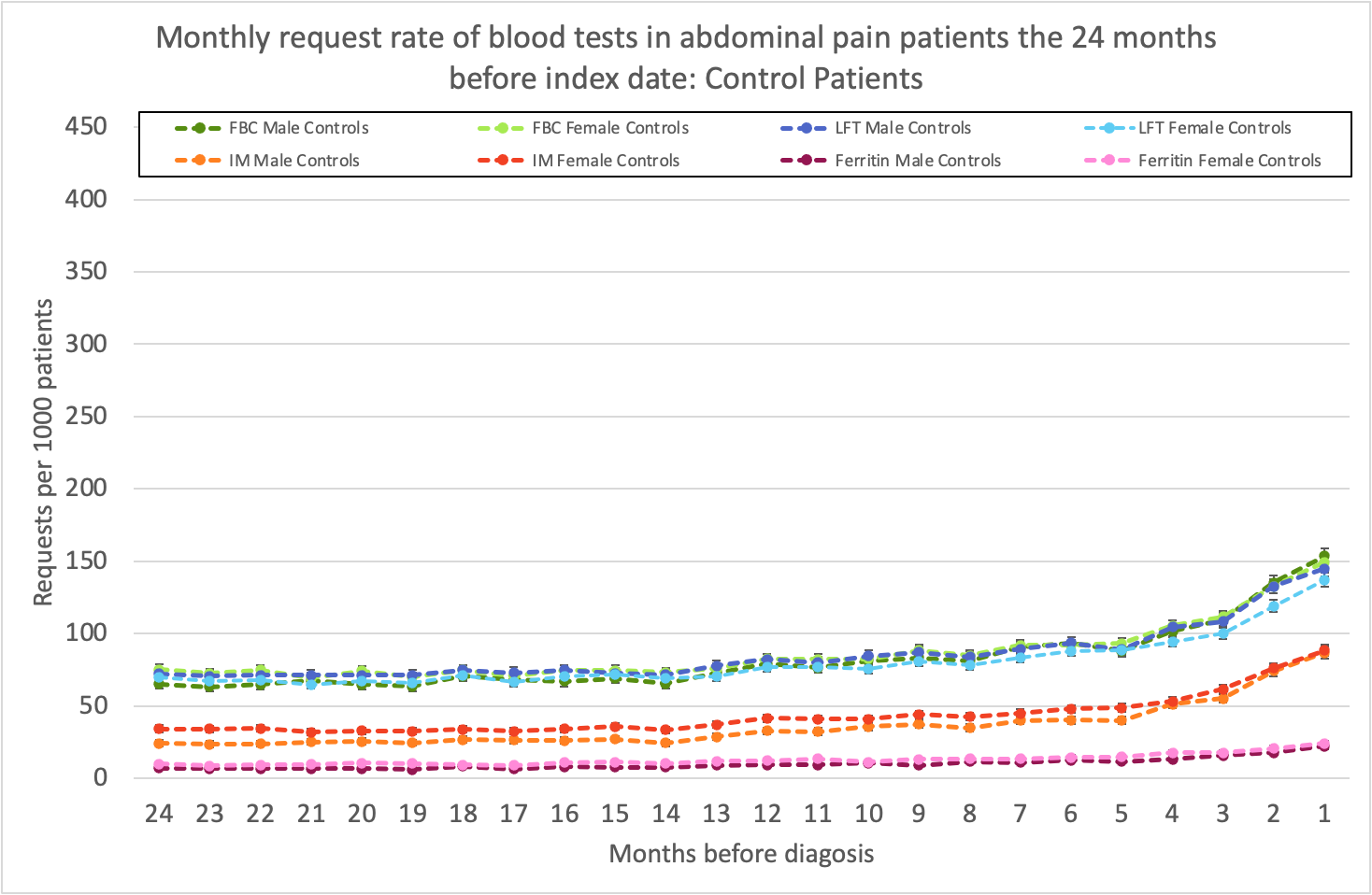  **B** |
| Abdominal Pain | 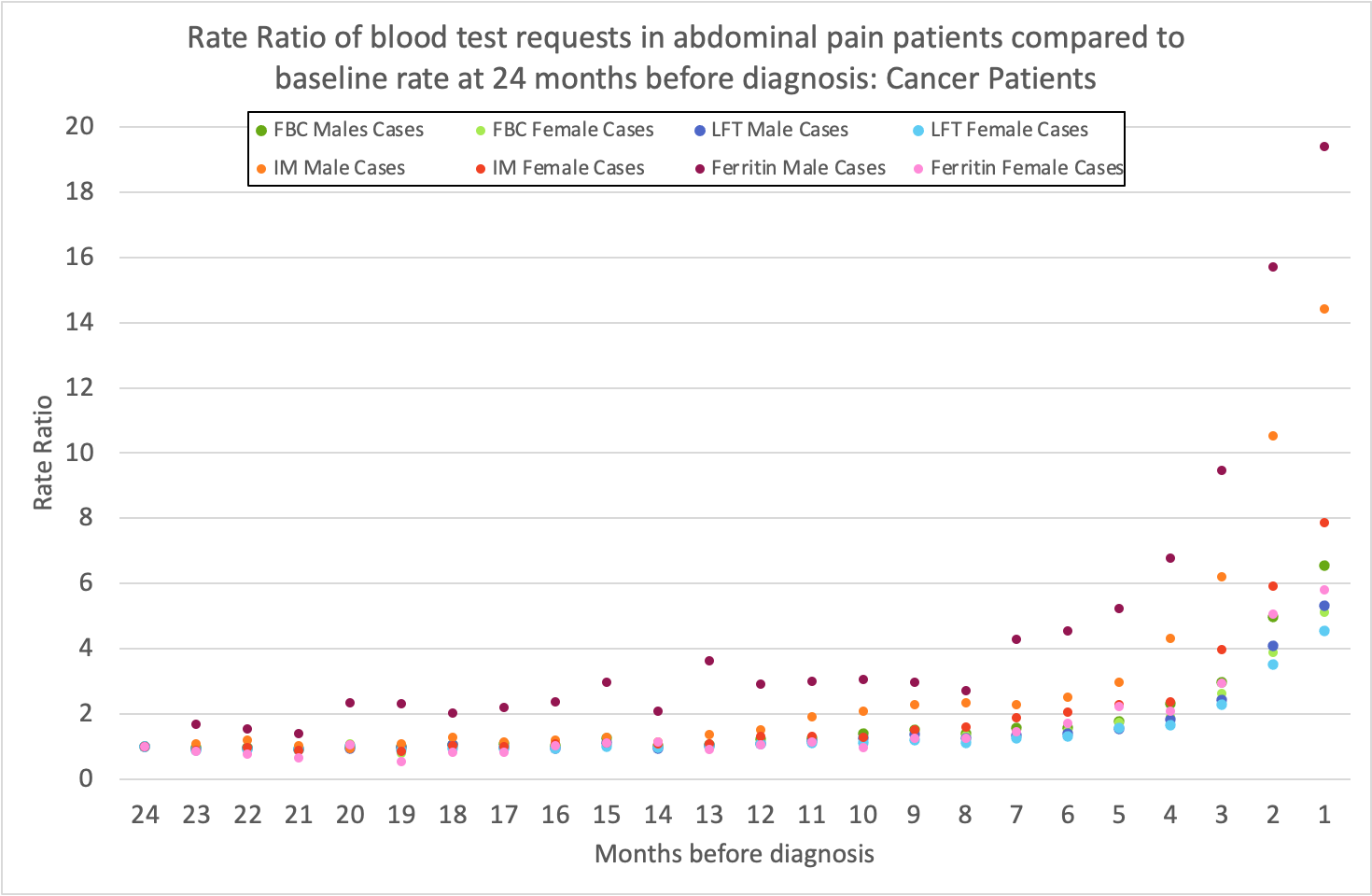  **C** | 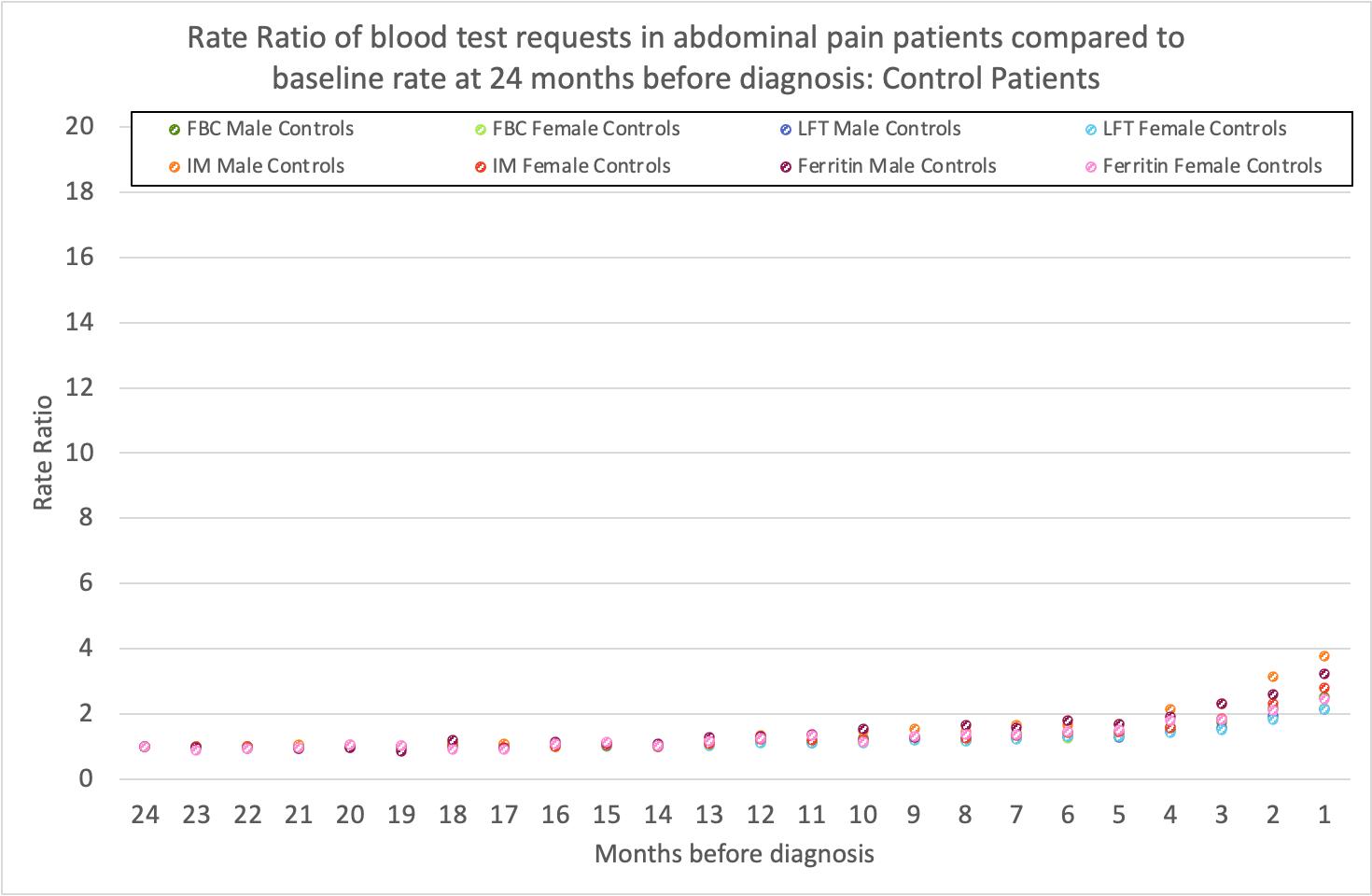  **D** |
| Abdominal Bloating | 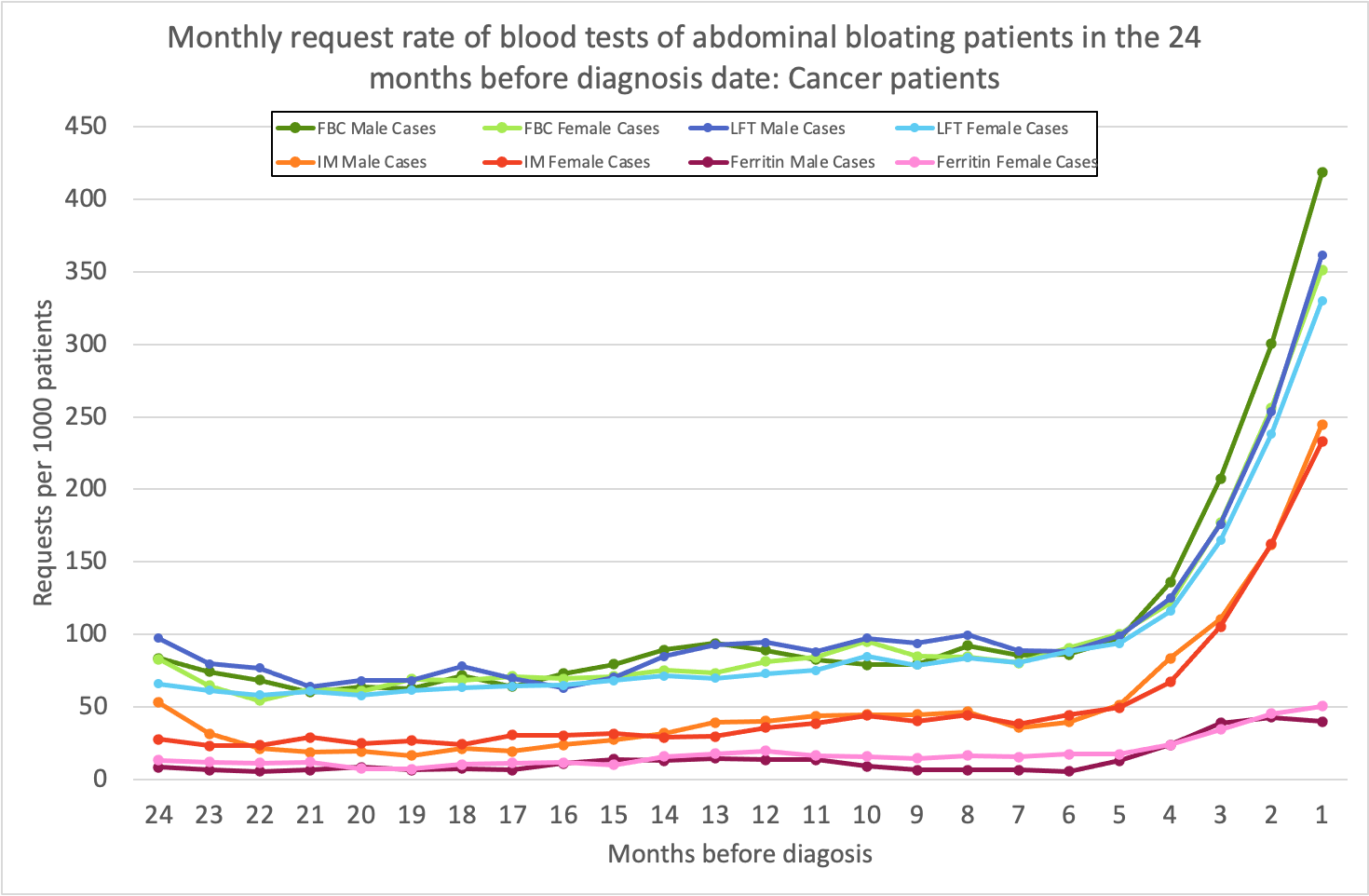  **E** | 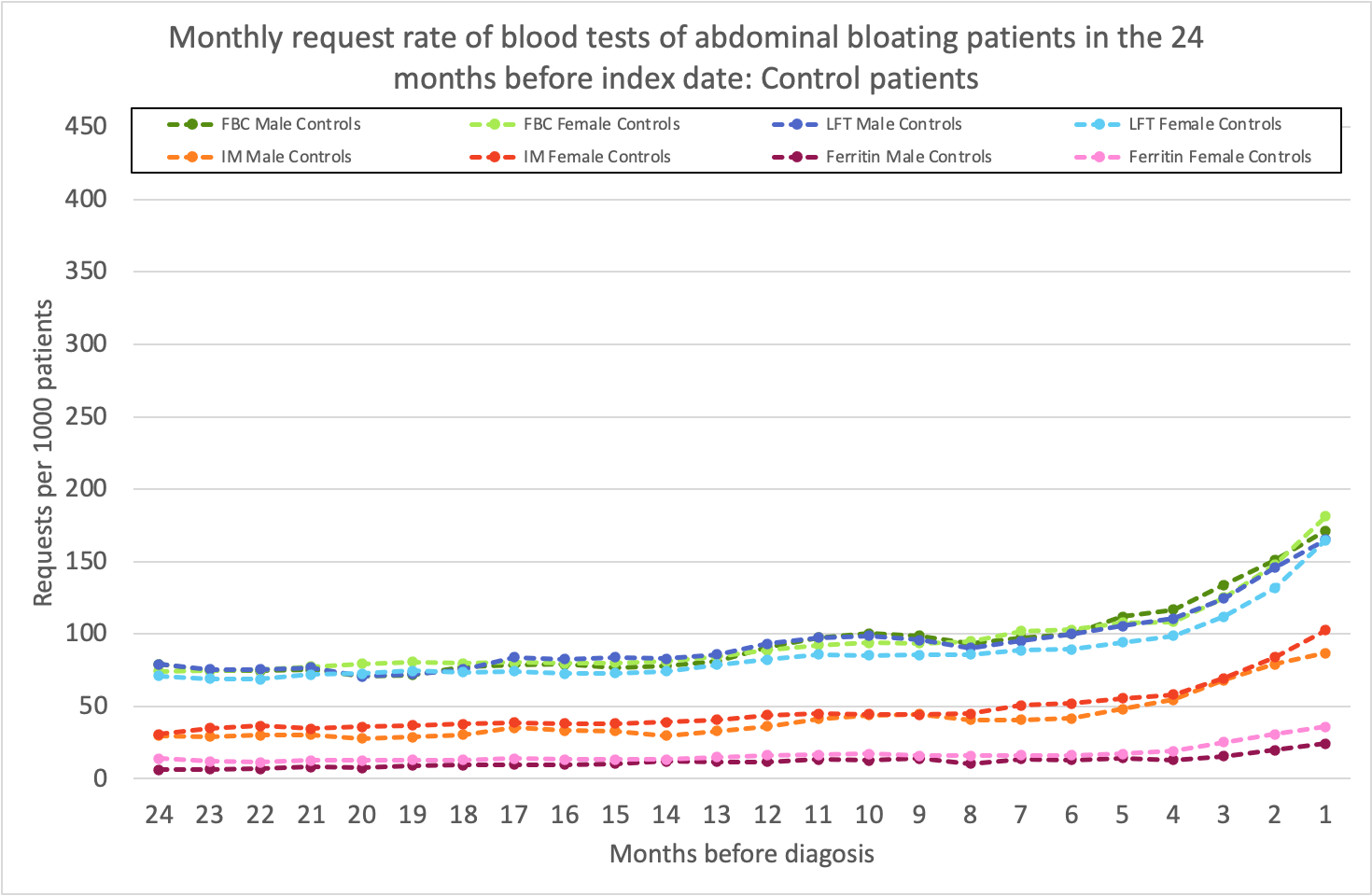  **F** |
| Abdominal Bloating | 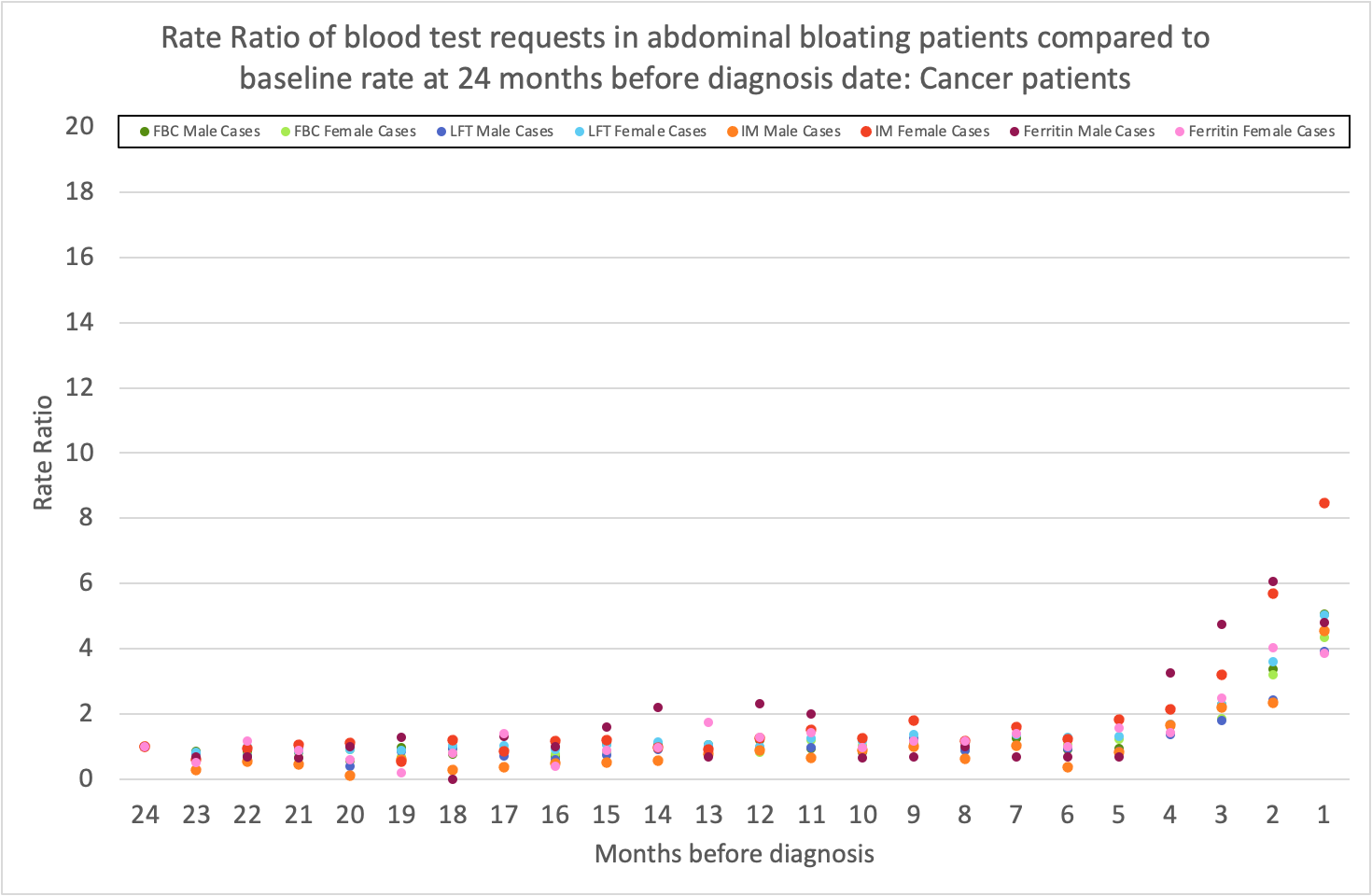  **G** | 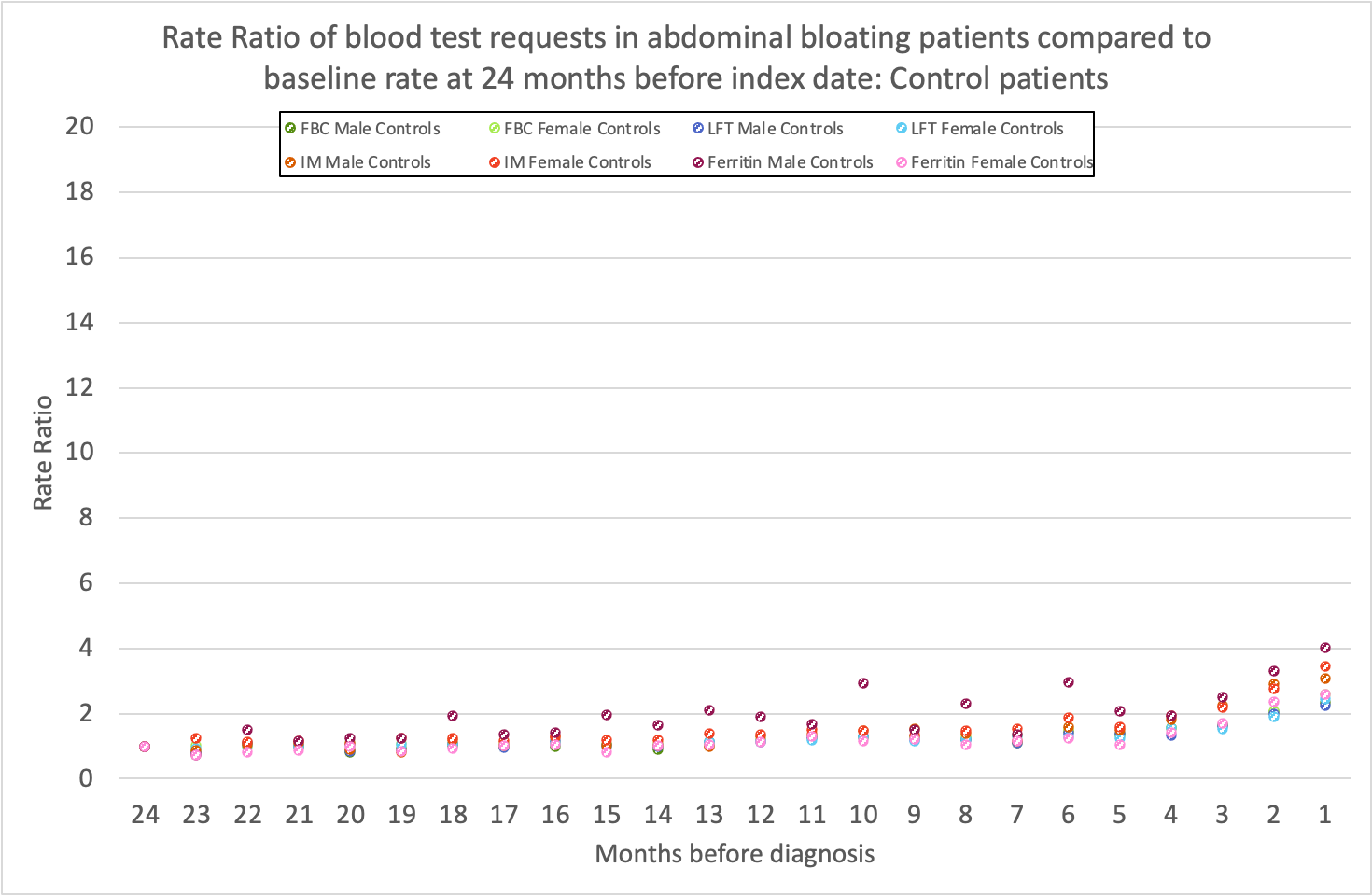  **H** |

##### Supplementary Figure S3: Monthly proportion of abdominal pain patients with an abnormal GP blood test in for the 24 months before diagnosis / index date comparing cases and controls.

Panels A, B, D and E: incident percentages, panels C and F: cumulative percentages over time. IM, inflammatory marker; WBC, total white blood cell count.

|  | Abdominal Pain (Males) | Abdominal Pain (Females) | Abdominal Pain (both sexes) |
| --- | --- | --- | --- |
| Cases | 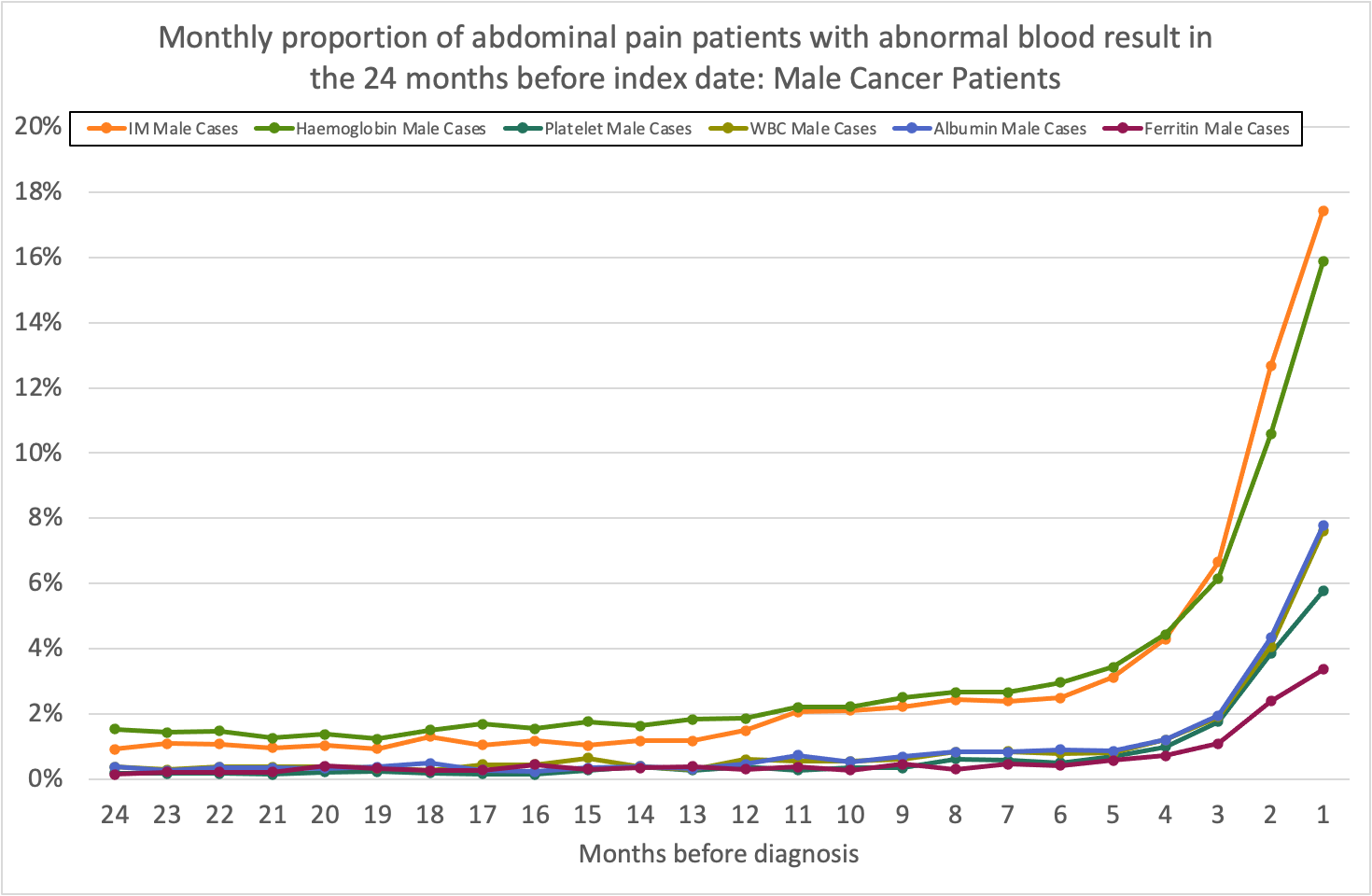  **A** | 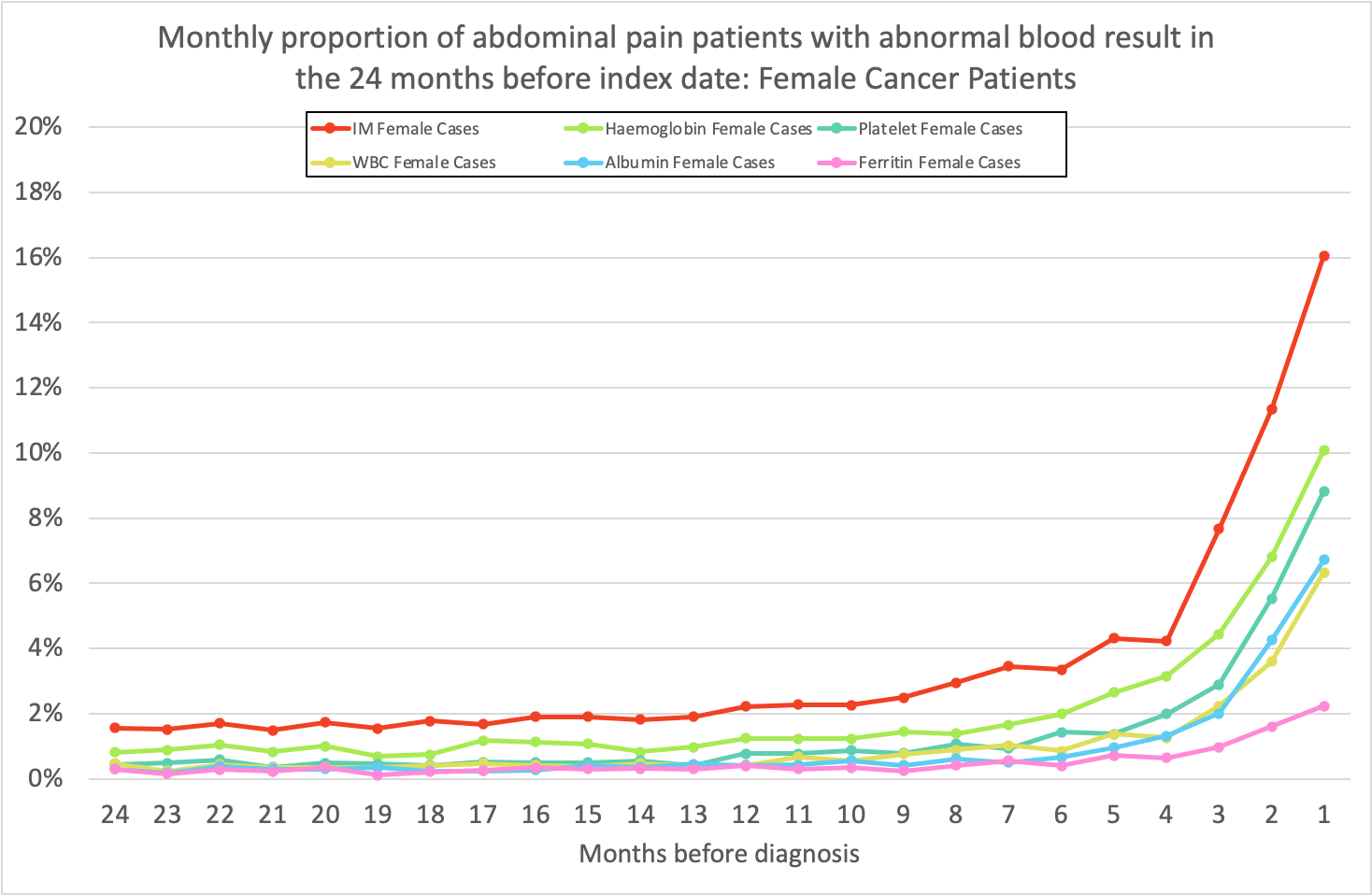  **B** | 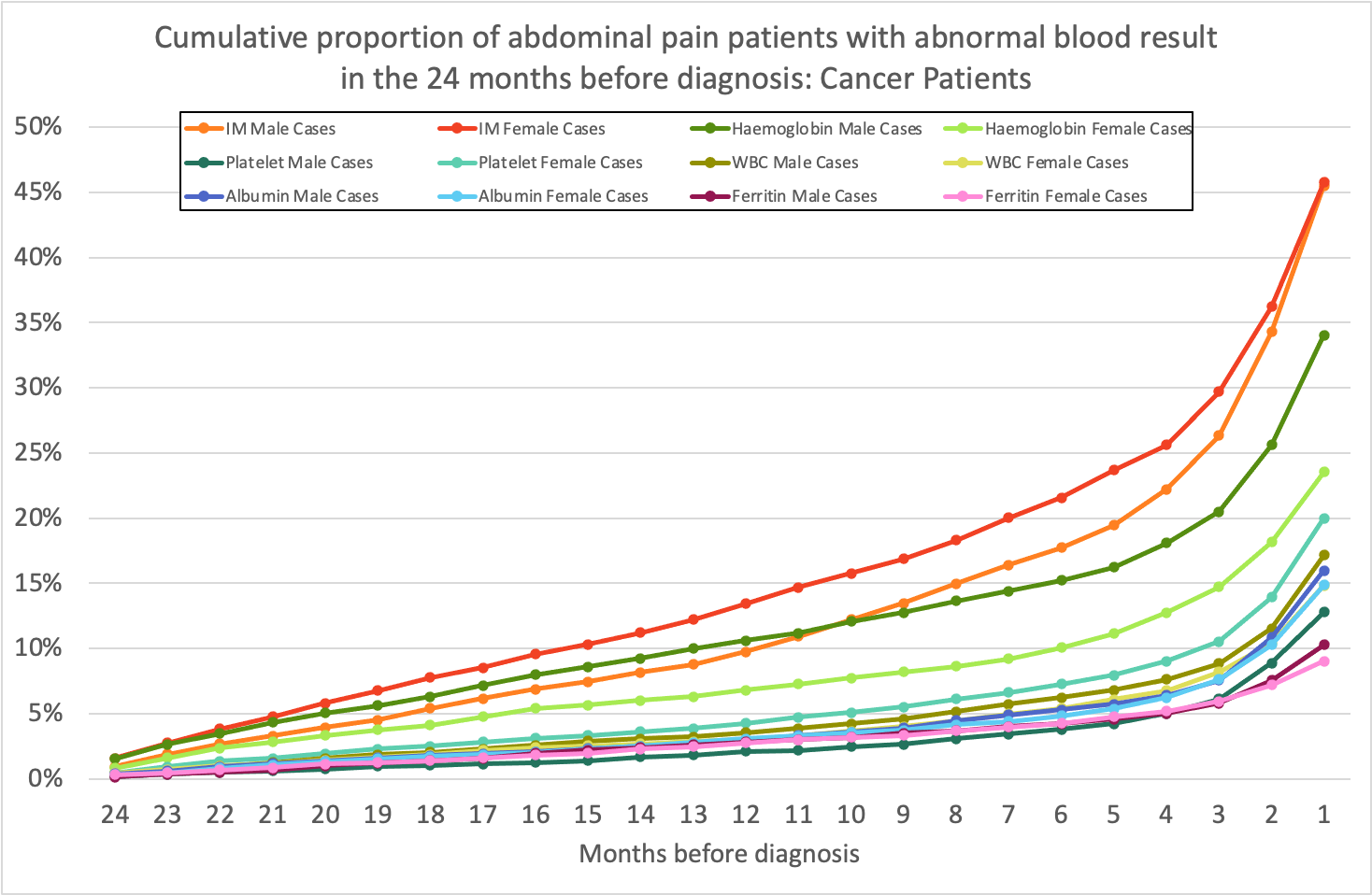  **C** |
| Controls | 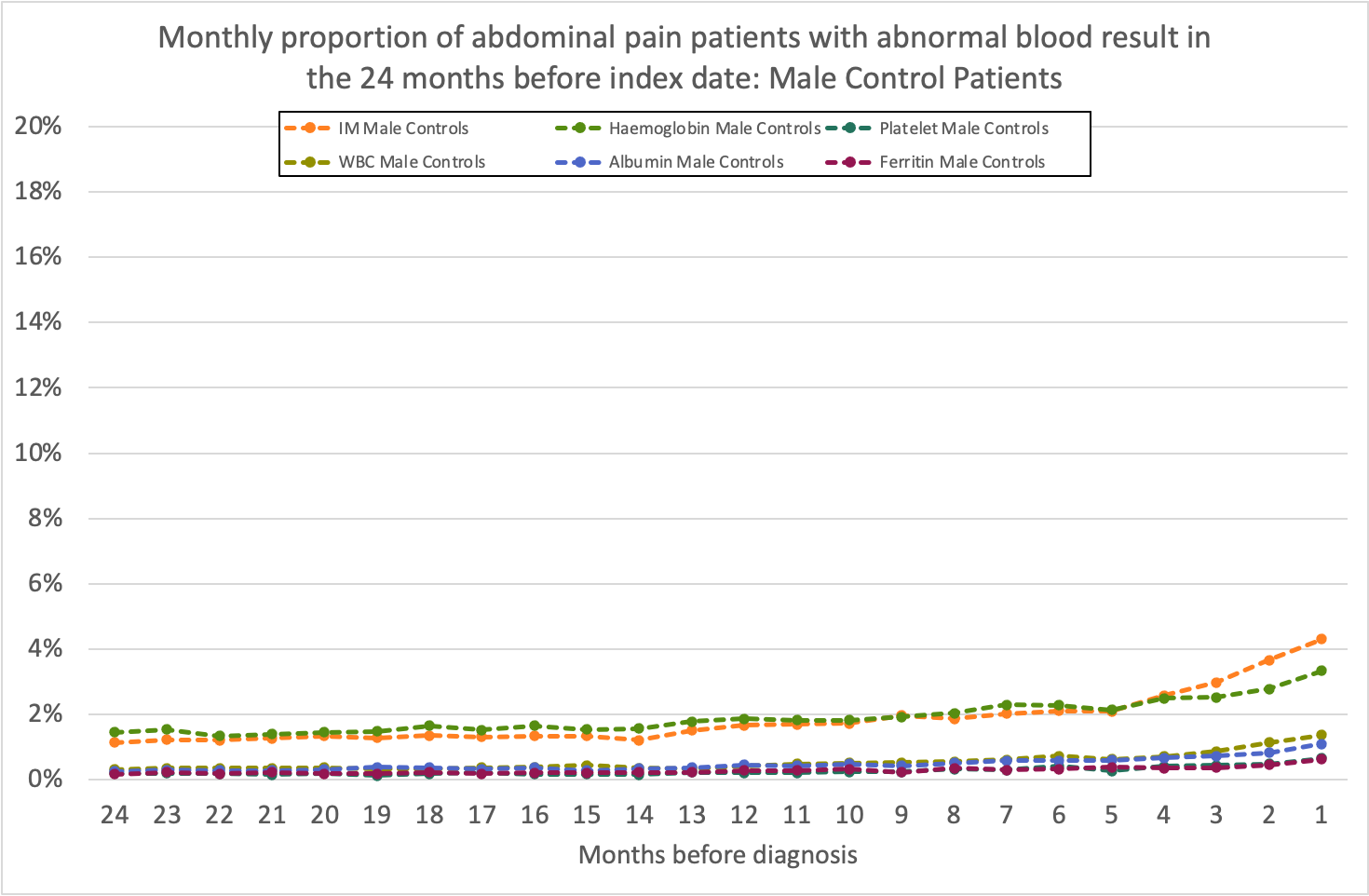  **D** | 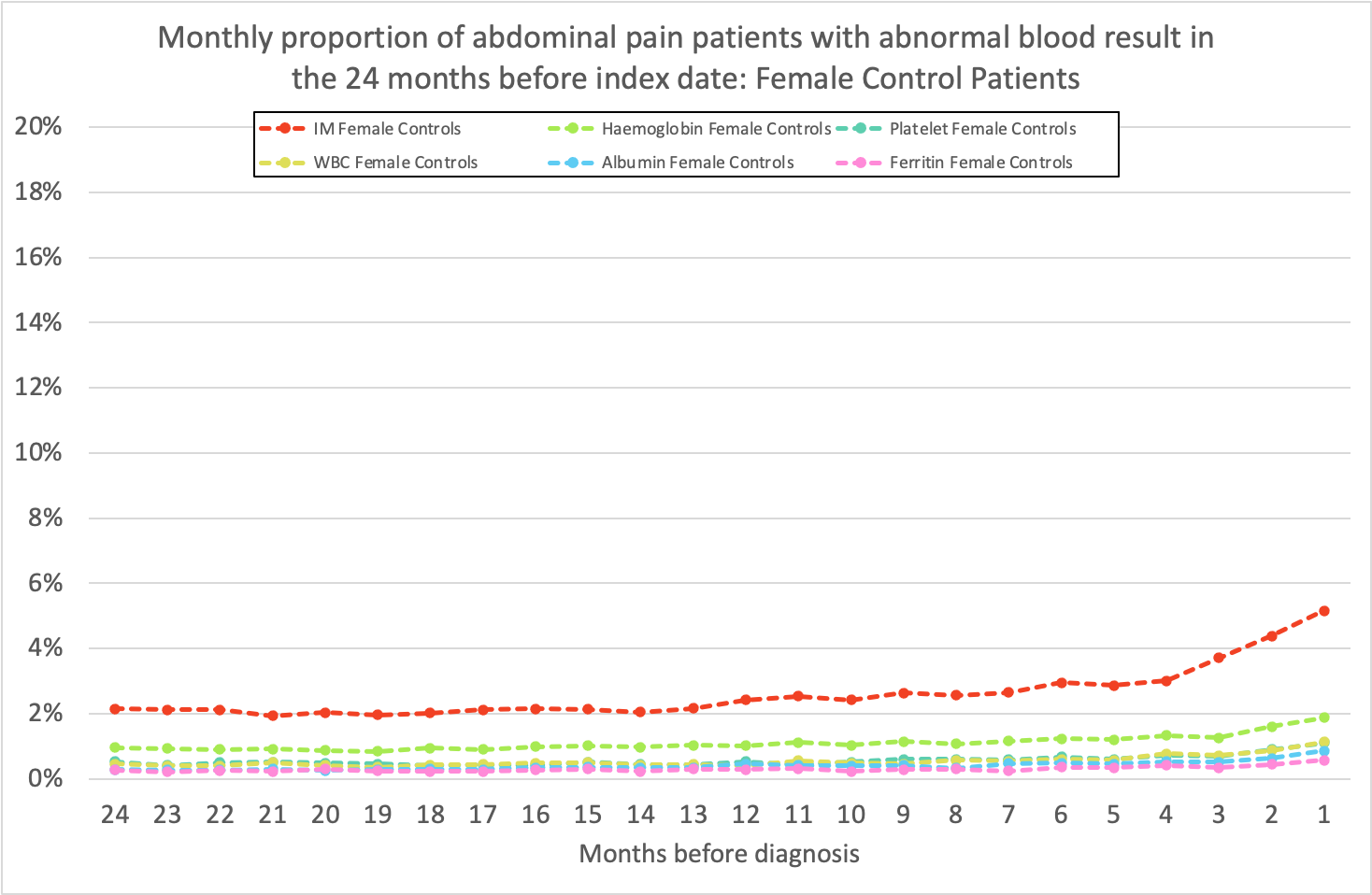  **E** | 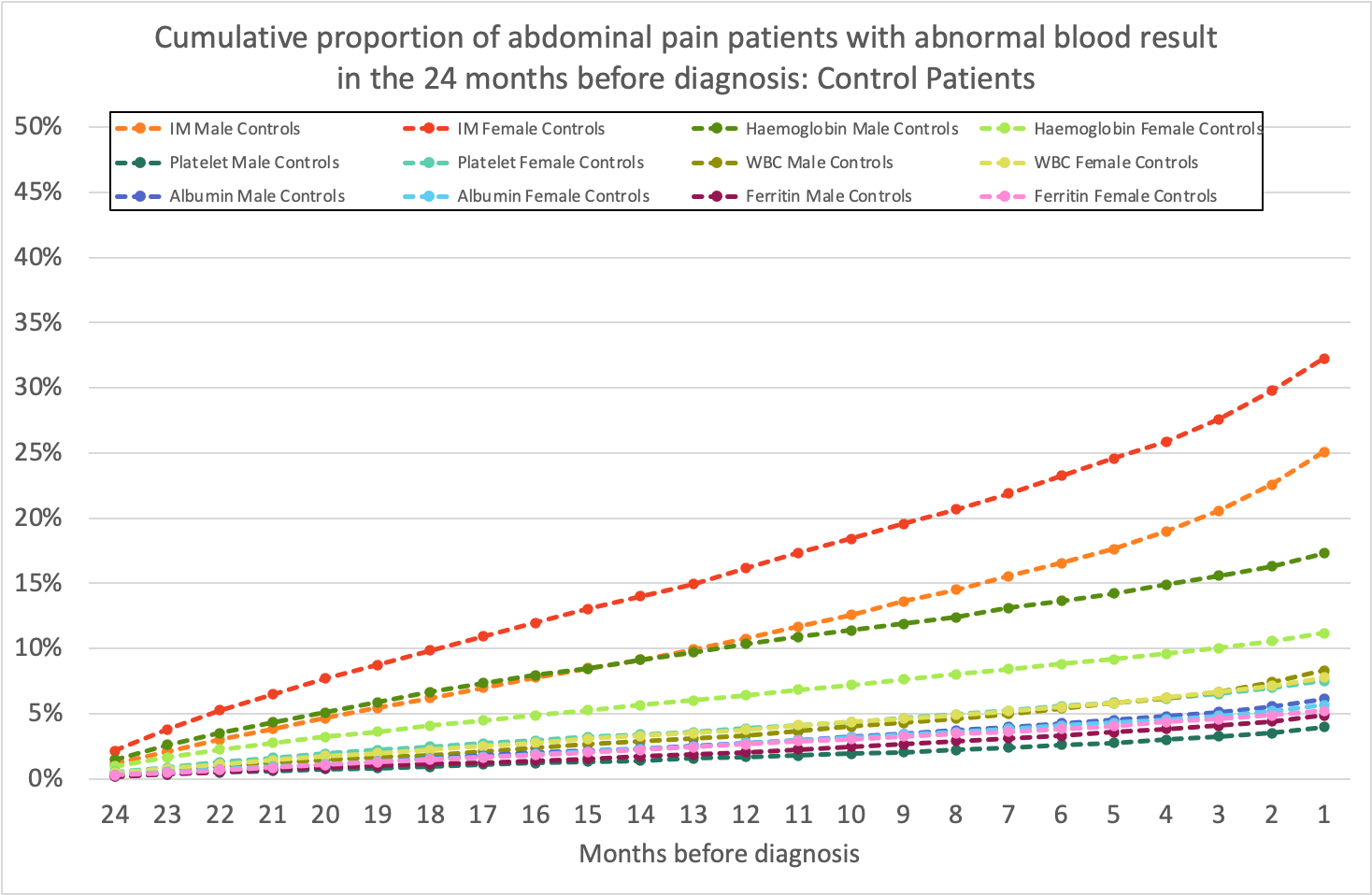  **F** |

##### Supplementary Figure S4: Monthly proportion of abdominal bloating patients with an abnormal GP blood test in for the 24 months before diagnosis / index date comparing cases and controls.

Panels A, B, D and E: incident percentages, panels C and F: cumulative percentages over time. IM, inflammatory marker; WBC, total white blood cell count.

|  | Abdominal Bloating (Males) | Abdominal Bloating (Females)  **C** | Abdominal Bloating (both sexes) |
| --- | --- | --- | --- |
| Cases | 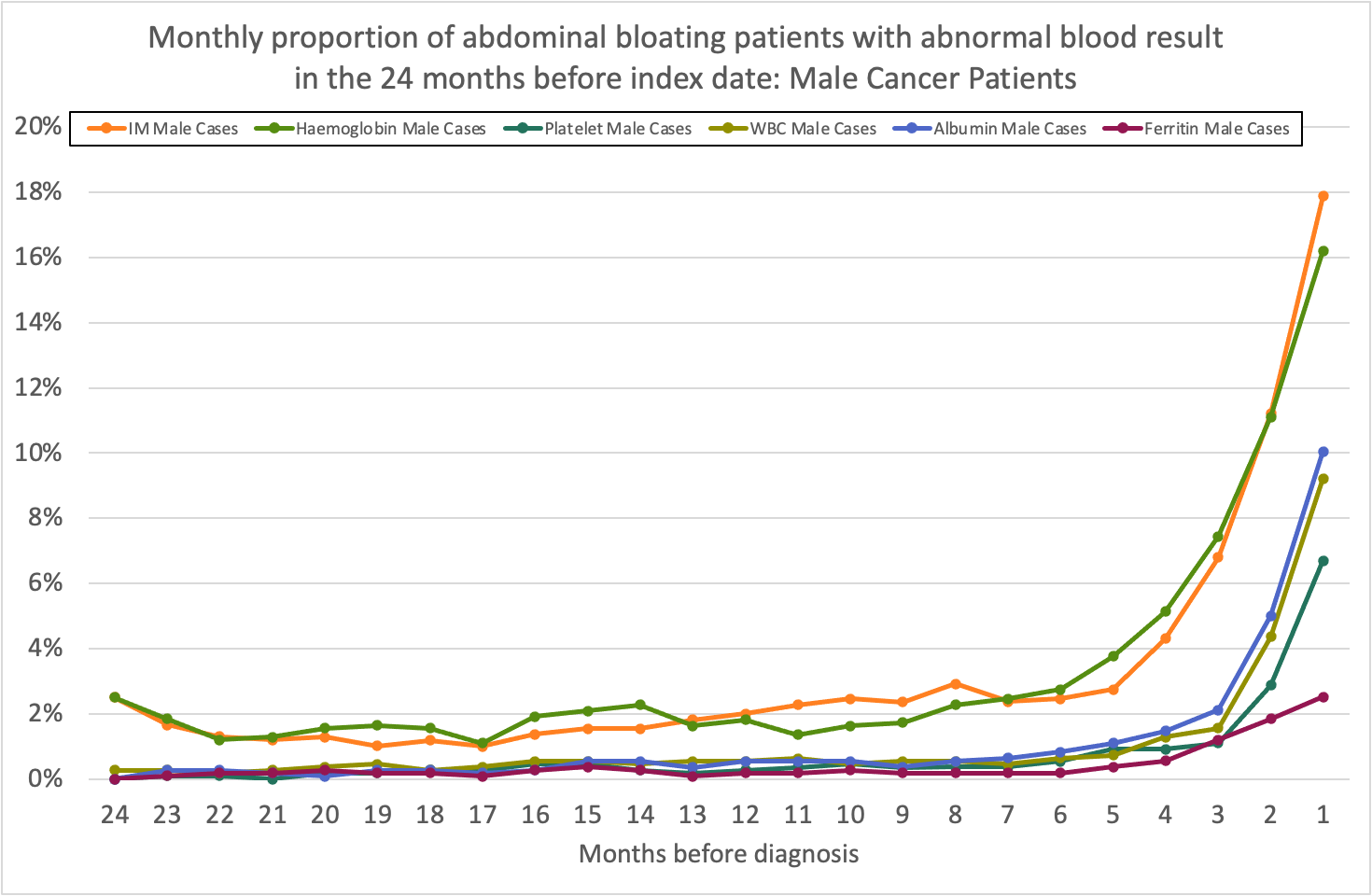  **A** | 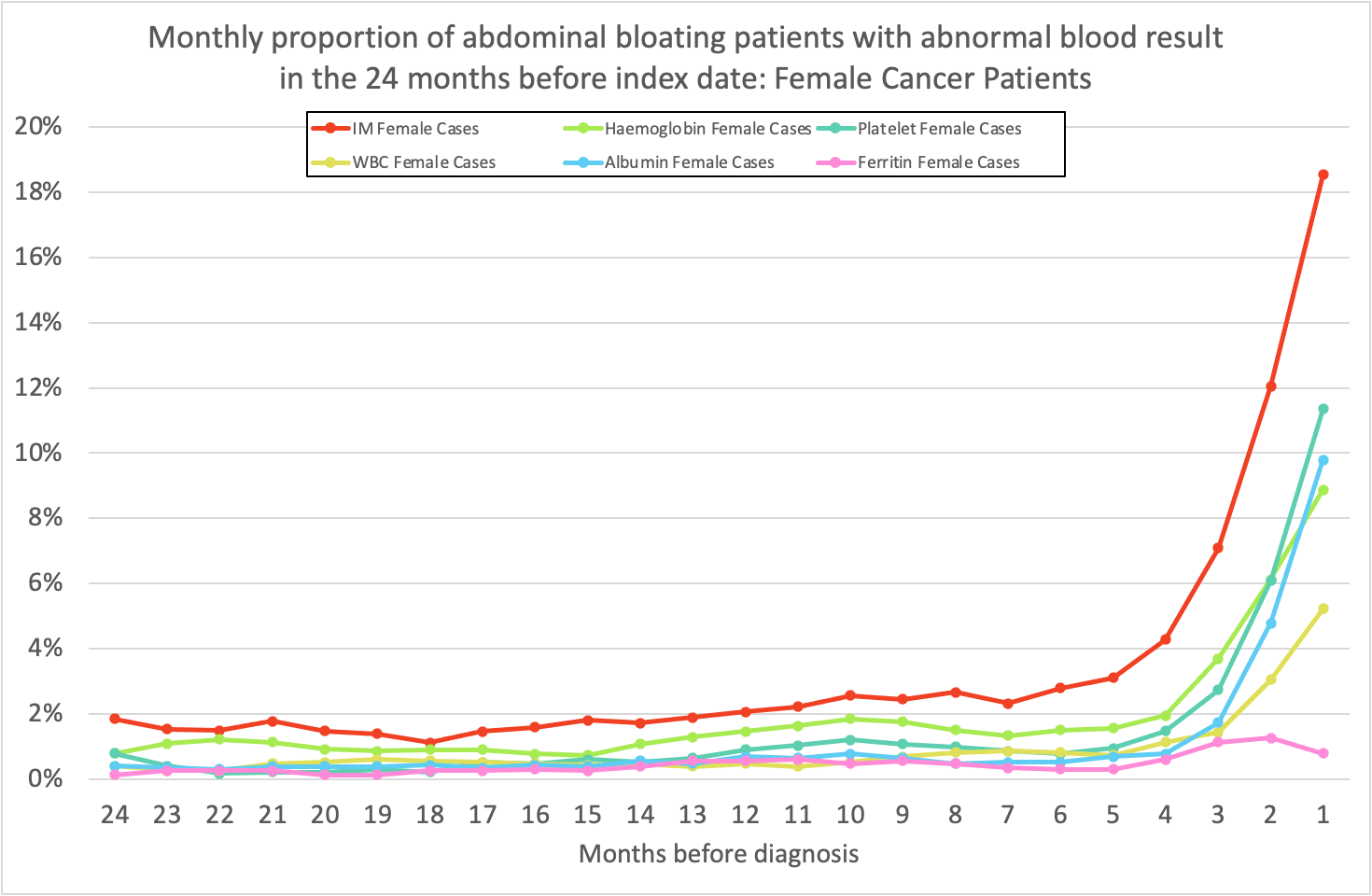  **B** | 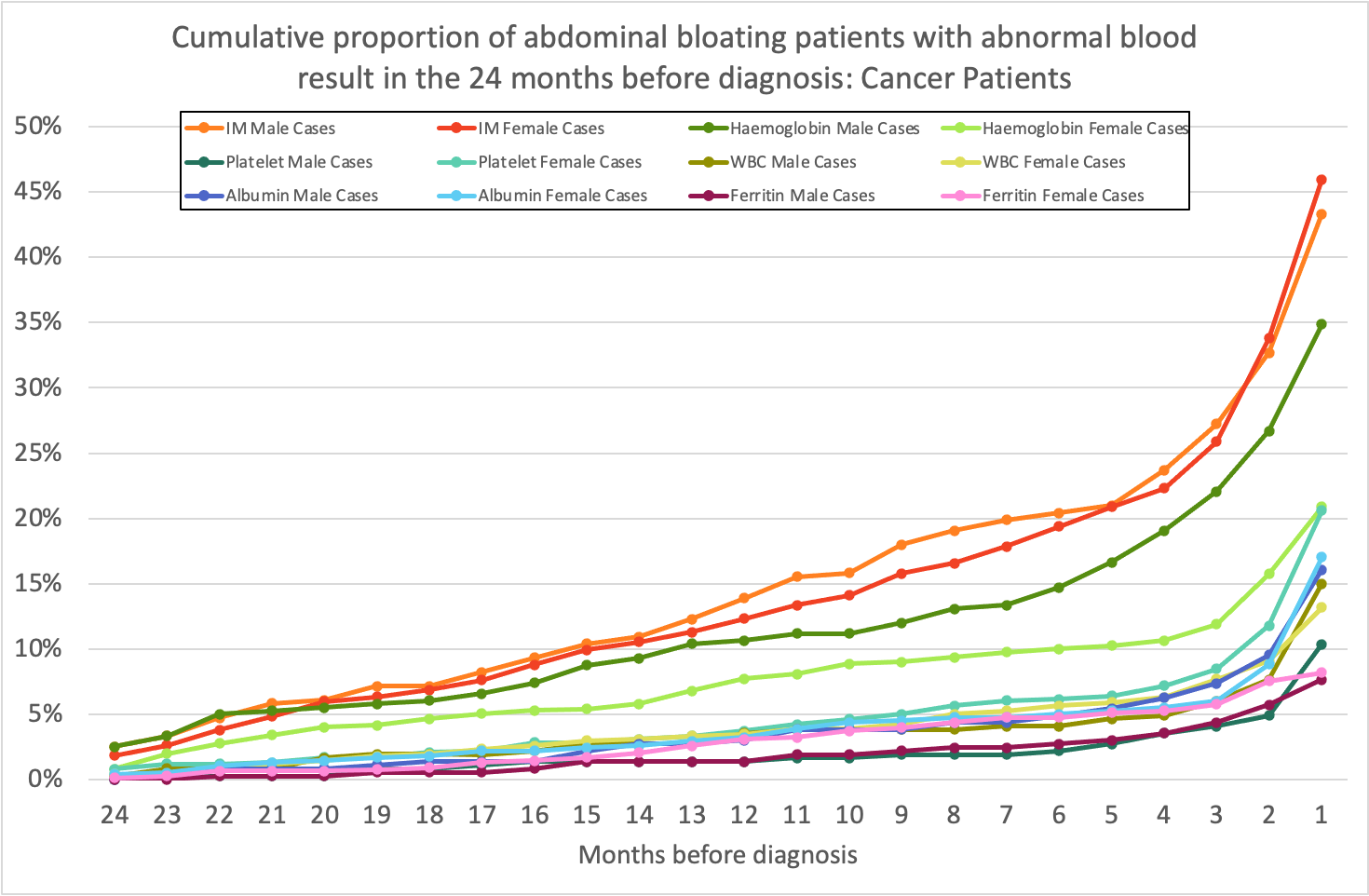 |
| Controls | 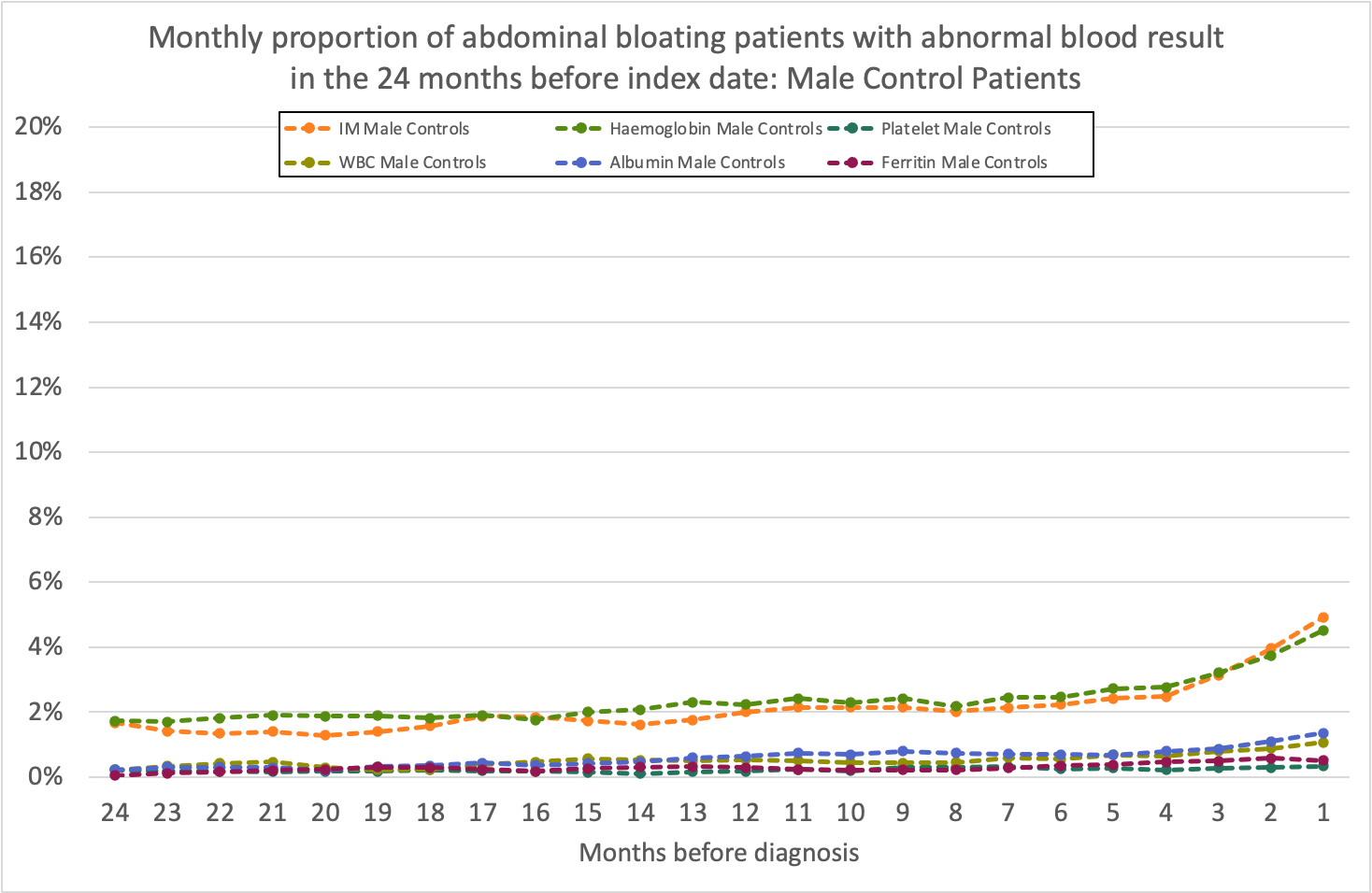  **D** | 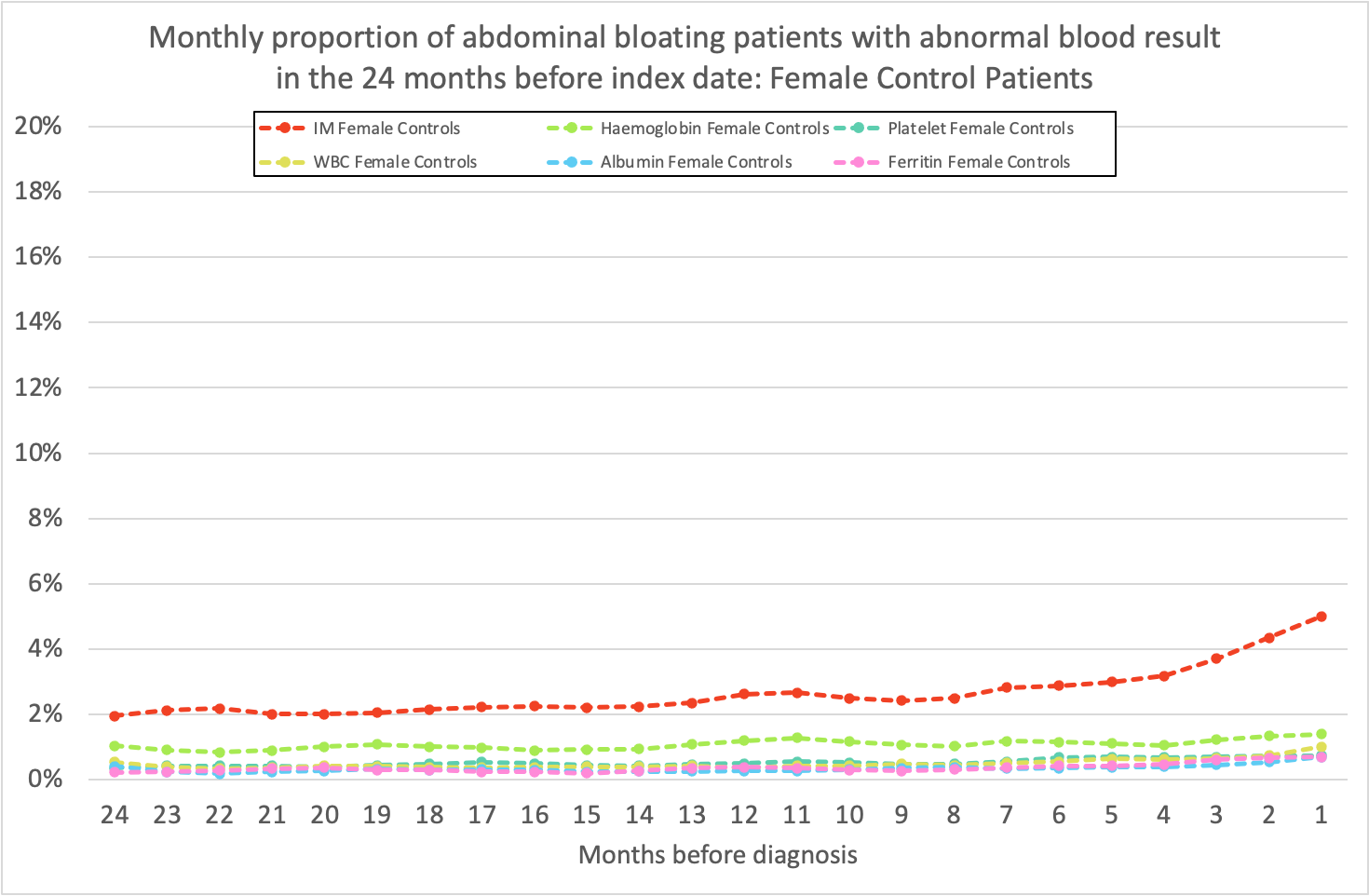  **E** | 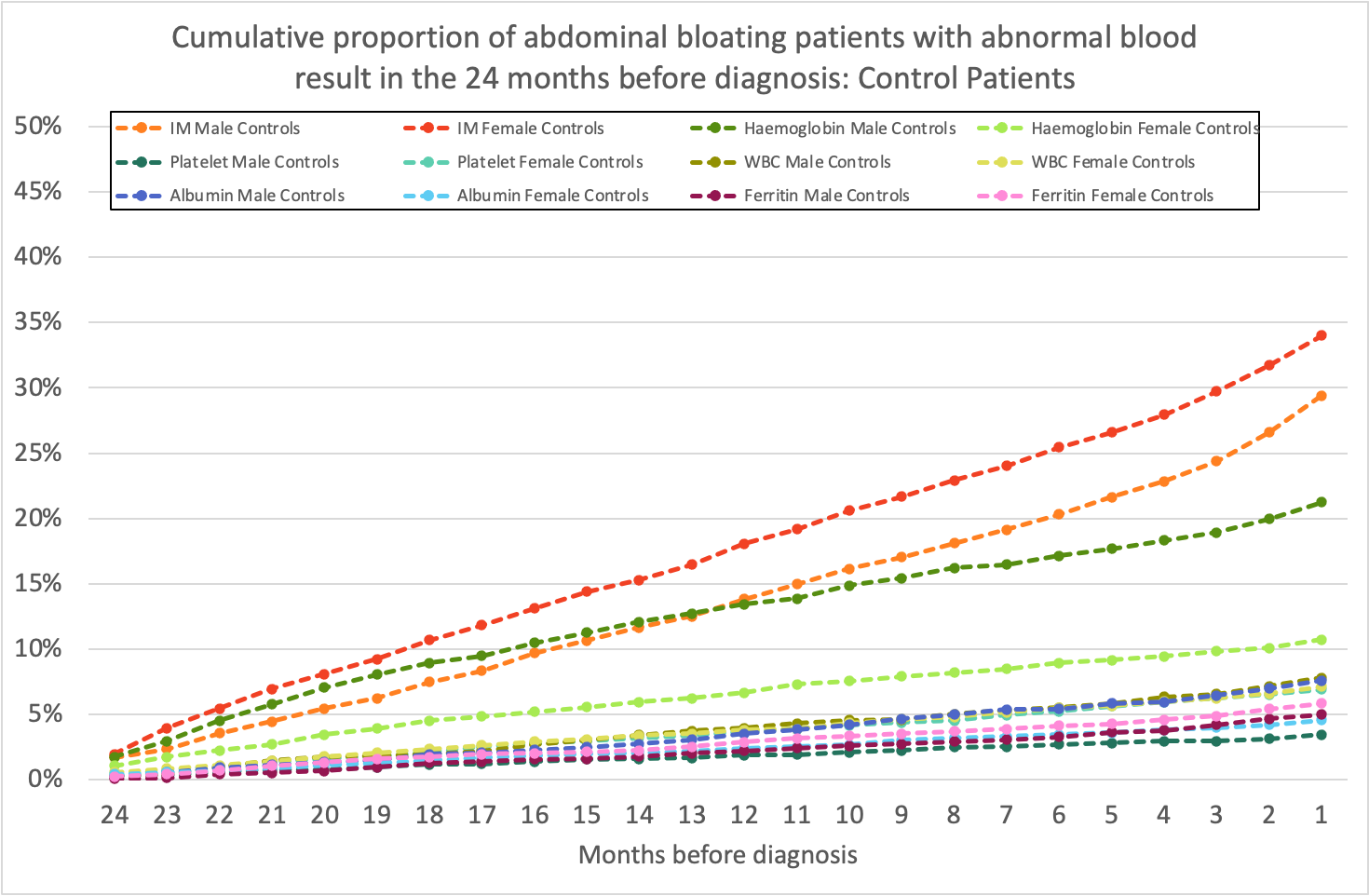  **F** |
